# Understanding the Behavior of Dicalcium Ferrite (Ca_2_Fe_2_O_5_) in Chemical Looping Syngas Production from CH_4_

**DOI:** 10.1021/acs.energyfuels.2c01065

**Published:** 2022-08-17

**Authors:** Made Santihayu Sukma, Yaoyao Zheng, Paul Hodgson, Stuart Ashley Scott

**Affiliations:** Department of Engineering, University of Cambridge, Trumpington Street, CB2 1PZ Cambridge, United Kingdom

## Abstract

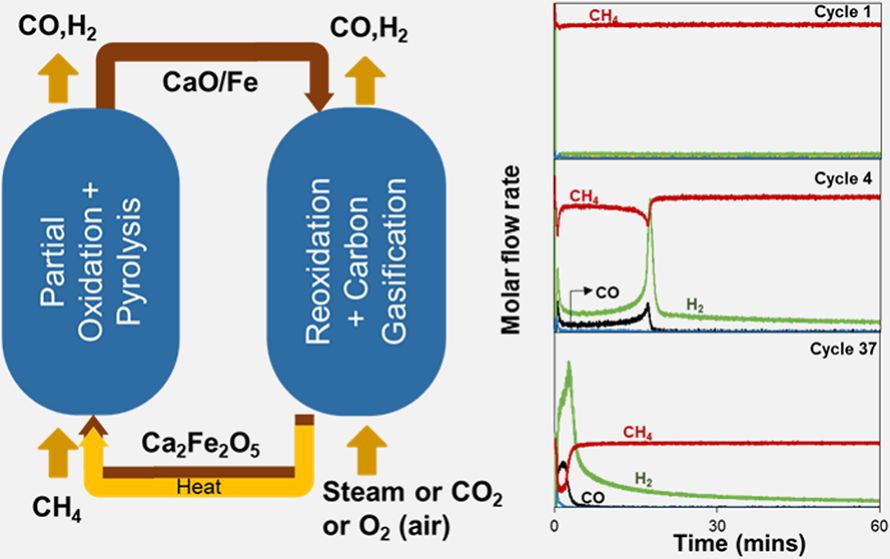

Previous work on calcium ferrites showed they were able to convert syngas to hydrogen via chemical looping. The mixture of iron and calcium and their oxides has different thermodynamic properties than iron oxide alone. Here, the use of methane, an abundant fuel, is investigated as the reductant in chemical looping syngas production. In contrast to syngas-fueled cycles, the looping materials became more active with cycling using methane as the fuel. When reduced by methane, the looping material often showed a significant induction period, indicating that products of reduction (in particular metallic Fe) acted as a catalyst for further reduction. The behavior in a thermogravimetric analyzer (TGA) and a fluidized bed was comparable, i.e., no degradation with cycling. The reduced C_2_F appeared to be easily reformed when oxidized with CO_2_, and there was little evidence of bulk phase segregation. The improved kinetics on cycling was likely due to the separation of metallic Fe onto the surface. Using hydrogen to partially reduce C_2_F promotes the catalytic pyrolysis of methane.

## 1 Introduction

Methane (CH_4_) is widely utilized to synthesize hydrogen, ammonia, methanol, and other higher hydrocarbons.^1^ It has the highest heat of combustion per CO_2_ emitted compared to other hydrocarbons.^2^ In 2019, approximately 95% of hydrogen produced was derived from natural gas or coal,^3^ and methane will remain a major feedstock for hydrogen production in the foreseeable future.^4,5^ Steam methane reforming (SMR) is the most common process used to convert methane to hydrogen;^3,4,6^ however, this process inherently emits a large amount of CO_2_ (9.5 kg-CO_2_/kg-H_2_).^3^

Methane can be partially oxidized into syngas (CO/H_2_), i.e.,  (Δ*H*_298 K_^°^ = −36 kJ mol^–1^). Partial oxidation of CH_4_ has a theoretical [H_2_]/[CO] ratio of 2, which is suitable for the gas-to-liquid process (GtL), i.e., via the Fischer–Tropsch (FT) process.^7−9^ When carried out homogeneously, very high temperatures are needed for partial oxidation; however, if a suitable catalyst is used (e.g., Ni, Fe, or noble metals), high selectivities toward H_2_ and CO can be achieved at much lower temperatures.^10,11^ Autothermal reforming (ATR) uses a combination of partial oxidation and steam-methane reforming in the same reactor to balance the heat load^12^ and directly produces the desired [H_2_]/[CO] ratio. For hydrogen production, partial oxidation and ATR would still require further steps to shift the CO product to H_2_ and to remove the carbon as CO_2_.

Chemical looping (CL) is an alternative approach to oxidation reactions, in which the oxygen transfer to a hydrocarbon like methane is mediated by a solid oxygen carrier, which first oxidizes or partially oxidizes the fuel and is then recharged with oxygen in a separate step, usually using air or steam.^13^ Unlike conventional partial oxidation, or ATR where pure O_2_ is required if N_2_ separation downstream is to be avoided, partial oxidation via chemical looping does not need an air separation unit.^14,15^ Here, the methane is oxidized, separately from where the oxygen in air (or steam) is reduced and the transfer is facilitated by a solid oxygen carrier, MeO_*x*_, which moves the oxygen between the different reactions, e.g.,Methane oxidation/oxygen carrier reduction
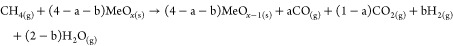
1

Air reduction/oxygen carrier regeneration
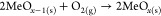
2

Steam reduction/oxygen carrier regeneration
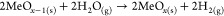
3

This has several potential advantages: (1) breaking the reaction into steps can reduce thermodynamic irreversibilities and allows heat to be extracted at temperatures of use to power cycles;^16^ (2) separations are performed inherently, in this case preventing N_2_ from the air either diluting the syngas or H_2_ products or CO_2_ (giving a built-in carbon capture system); and (3) varying the extent of oxidation can balance the heat loads between the different stages. The selectivity of methane oxidation toward partial combustion vs complete combustion can be tailored by selecting suitable materials for the oxygen carrier. The tendency of an oxygen carrier to perform partial oxidation vs complete combustion of methane depends on its thermodynamic properties and in particular the equilibrium partial pressure of oxygen (*P*_O_2__) for the phase transition being utilized.^17^ For example, copper-based metal oxides are attractive for chemical looping combustion (CLC) applications where complete oxidation is desired, due to their high *P*_O_2__.^18−20^ On the other hand, oxygen carriers with sufficiently low *P*_O_2__ have been investigated for hydrogen production using steam–known as chemical-looping water splitting (CLWS). The low *P*_O_2__ of these oxygen carriers implies that the reduced metal oxides can be oxidized with steam to produce hydrogen or with CO_2_ yielding CO.^21−27^ Chemical looping water splitting was initially introduced in 1913 using iron oxides in the steam-iron process.^28^ The low value of *P*_O_2__ of the metal oxides used for water splitting also means that they tend toward partial oxidation over complete combustion and, hence, are selective toward syngas.

The concept of utilizing materials with low *P*_O_2__ to produce syngas has recently gained popularity,^1,15,29^ including the use of more complicated, nonstoichiometric, perovskite-based oxygen carriers such as La_*x*_Sr_1–*x*_Fe_*y*_Co_1–*y*_O_3−δ_,^30^ which has a high selectivity toward syngas, and La_0.85_Sr_0.15_Fe_0.95_Al_0.05_O_3−δ_, which was able to produce almost pure syngas.^14^ Iron-based oxygen carriers are particularly attractive in this application since they are abundantly available from natural precursors such as iron ores; hence, cost is low, and the materials are not hazardous. However, pure iron oxide deteriorates severely after just a few cycles, especially if it is reduced into metallic iron;^31^ therefore, suitable supports are essential. Previous works showed calcium oxide (CaO) is a promising support material for Fe_2_O_3_ in chemical looping applications, due to the material’s robustness in cyclic experiments.^22−24^ CaO and Fe_2_O_3_ form different mixed phases of calcium ferrites, i.e., Ca_2_Fe_2_O_5_ (**C**_**2**_**F**) and CaFe_2_O_4_ (**CF**), and so the support material, while not undergoing redox, is also not entirely inert. Calcium ferrites have very different thermodynamic properties to pure iron oxides. This has previously been exploited to increase the equilibrium conversion of steam in water splitting.^22,24,32^ Calcium ferrites are remarkably stable in cycles when reduced in CO and replenished using CO_2_, compared with unsupported iron oxide which shows a declining performance.^22^ Calcium ferrites’ ability to generate hydrogen by replenishing the reduced metal oxides using steam has been widely studied.^22−25,33^ However, only a few studies, e.g., Sun et al.^33^ and Hosseini et al.,^25^ have examined the use of methane as the reductant in the application of chemical looping with calcium ferrites.

In the presence of reduced phases in these chemical looping systems, methane can also pyrolyze and deposit carbon. In fact, iron is a known catalyst for methane decomposition.^4,6,34^ Supported (e.g., Al_2_O_3_,^6,34^ CeO_2_,^35^ MgO^36^) iron catalysts have been evaluated for methane decomposition into solid carbon and hydrogen. This was also observed from the reduced perovskite oxide containing Fe (i.e., La_0.5_Sr_0.5_Fe_0.5_Co_0.5_O_3−δ_).^30^ Methane pyrolysis may therefore play an important role in the interaction of carbon with the metal oxide, either beneficially, e.g., where the methane is deliberately decomposed into carbon on the surface to produce hydrogen or as part of the partial oxidation mechanism,^11^ or deleteriously, e.g., when coke buildup hinders the reaction. Any coke buildup will also contaminate the regeneration steps with carbon reducing the purity of hydrogen and resulting in CO_2_ emission.^31^

**Figure 1:**
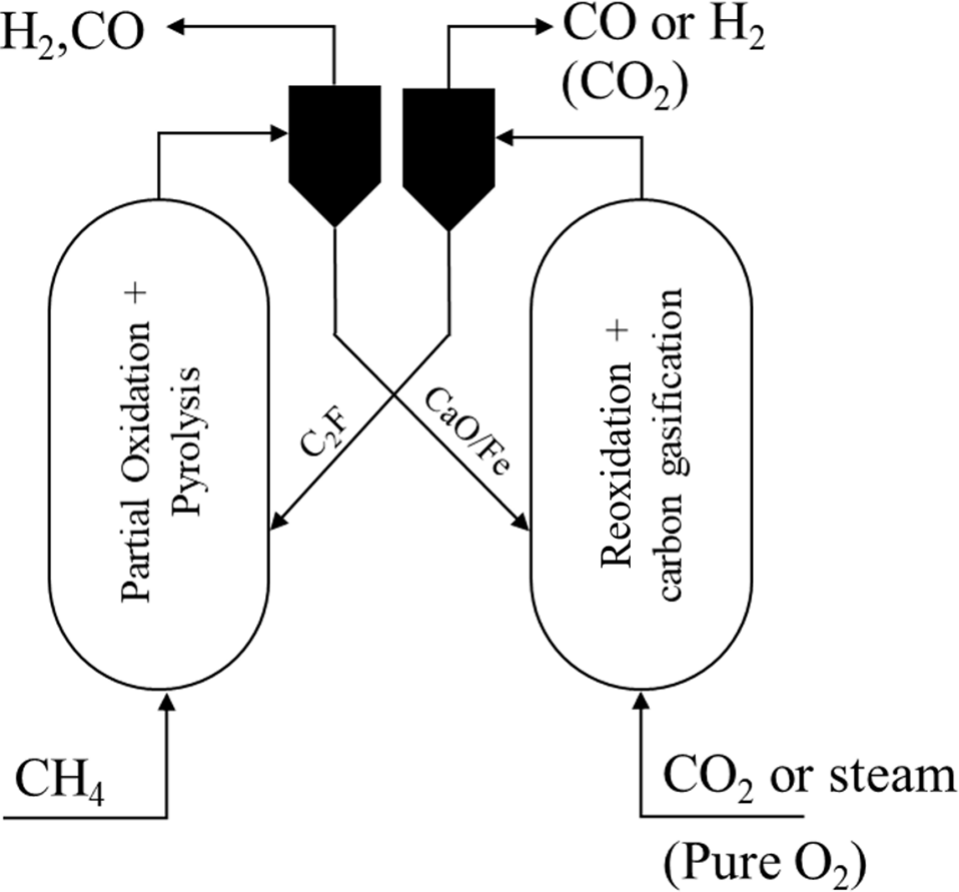
Integrated partial oxidation and pyrolysis of methane.

In this work, we propose a chemical looping process that integrates partial oxidation and pyrolysis of methane in chemical looping syngas production, using a Ca–Fe–O oxygen carrier, dicalcium ferrite (Ca_2_Fe_2_O_5_, **C**_**2**_**F**). Figure 1 shows a schematic diagram of the proposed system. C_2_F first transfers its lattice oxygen to partially oxidize methane, i.e.,  ⇋  +  (Δ*H*_298 K_^°^ = +253 kJ mol^–1^). In reduced C_2_F, iron is fully reduced to Fe^0^, which is a catalyst for methane pyrolysis.^6,15,30^ Methane pyrolysis,  ⇋ C_(s)_ +, is less endothermic than the partial oxidation by C_2_F, i.e., Δ*H*_298 K_^°^ = of 75 kJ mol CH_4_^–1^ (from MTDATA/sub-sgte database^37^); therefore, combining partial oxidation and pyrolysis of CH_4_ could potentially reduce the energy requirement in the partial oxidation reactor. If steam or CO_2_ were used as the oxidant, any solid carbon would be gasified during the regeneration, thus generating more H_2_ or CO. Generation of the CO during regeneration with CO_2_ may or may not be desirable depending on whether hydrogen or syngas is the desired end product. Alternatively, the carbon could be removed by combustion in air (or oxygen if full carbon capture is required), i.e., C_(s)_ +  → , generating more heat overall. While carbon gasification in CO_2_ is endothermic, Δ*H*_298 K_^°^ = +172 mol C^–1^, carbon combustion in O_2_ is very exothermic, i.e., Δ*H*_298 K_^°^ = −394 kJ mol C^–1^.

Figure 1 shows the regeneration of the oxygen carrier as only a single stage, fed by either gasifying agent (CO_2_ or H_2_O) or oxidant (O_2_). However, it is possible to vary the extent of total combustion of the methane by either mixing the gasifying agent with O_2_ or breaking the regeneration into multiple stages, i.e., oxidation in H_2_O/CO_2_ followed by oxidation in air/O_2_. Oxidation with CO_2_ can fully replenish the reduced C_2_F, i.e.,  ⇋ (Δ*H*_298 K_^°^ = −6.8 kJ mol^–1^) and/or steam, i.e.,  ⇋  (Δ*H*_298 K_^°^ = −48 kJ mol^–1^) to produce CO and/or H_2_, respectively, in addition to gasifying any carbon. Overall, the partial oxidation of methane by the oxygen carrier and its subsequent oxidation in CO_2_ or steam is equal to dry reforming of methane (DRM) or steam methane reforming (SMR), respectively.^14^ Overall heat balance can be achieved using air (or O_2_) for some of the oxidation in  ⇋ , which is extremely exothermic with Δ*H*_298 K_^°^ = −290 kJ mol^–1^. Thus, there is considerable flexibility by varying the extent of carbon deposition in the methane conversion stage, and the relative amount of oxidation carried out by CO_2_/H_2_O vs O_2_. In this way, the syngas ratio can be adjusted in accordance with the requirement of subsequent processes, e.g., for GtL processes.

Here, the use of CH_4_ as the fuel to reduce C_2_F was studied in a thermogravimetry analyzer (TGA) and a fluidized bed. A number of cycles of (i) CH_4_ reduction, (ii) CO_2_ oxidation, and (iii) air oxidation were performed to investigate the cyclability of C_2_F. Previous studies reported that C_2_F has poor kinetics when it is reacted with CH_4._^25,33^ Coking on the C_2_F surface and its impact on the performance of the metal oxide carrier were also explored.

## 2 Experimental Section

### 2.1 Materials Synthesis

Ca_2_Fe_2_O_5_ (**C_2_F**) was synthesized by mechanical mixing in a ball mill. Measured amounts of Fe_2_O_3_ (iron(III) oxide, 98%, 325 mesh powder, Thermo Fisher Scientific) and CaCO_3_ (calcium carbonate precipitated, Fisher Scientific) were mixed together with deionized (DI) water to obtain a molar ratio of  of 0.5. Ten wt % potato starch (BDH Laboratory Supplies) was added to the materials to improve the microporous structure of the particles. The powders were mixed in the ball mill for 3 h at 25 Hz. The resulting materials were then dried overnight in the oven at 100 °C before being calcined at 1000 °C for 6 h. The calcined materials were then crushed and sieved to 355–500 μm for thermogravimetric analysis (TGA) and 500–800 μm for fluidized bed experiments. Unsupported Fe_2_O_3_ was prepared using agglomeration; Fe_2_O_3_ powder was mixed with 10 wt % potato starch using a kitchen mixer. DI water was continuously sprayed, while the mixture was being stirred to generate agglomerates. These agglomerates were then sieved to obtain particle size in a range of 355–500 μm and then dried in an oven at 100 °C overnight. The dried particles were then calcined in a furnace at 900 °C for 2 h and resieved to obtain the desired particle size.

### 2.2 Thermogravimetric Analysis (TGA)

Temperature-programmed reduction (TPR) from 200–900 °C and isothermal reduction–oxidation (redox) cycles at 900 °C using 5% CH_4_ or 5% H_2_ balance N_2_ (50 mL/min at 20 °C and 1 atm) were performed in a TGA (TGA/DSC 1, Mettler Toledo). N_2_ gas was constantly supplied to the system as protective and purging gases, both at a flow rate of 50 mL/min (at 20 °C and 1 atm) during all TGA experiments. Prior to TPR experiments, materials were dried to remove any absorbed CO_2_ and moisture by putting around 20–40 mg of samples in alumina crucibles, ramping the temperature up to 900 °C at a rate of 20 °C/min, and holding it for 30 min in dried air. Subsequently, the materials were cooled down under N_2_, and the TPR was performed by heating up materials from 200–900 °C at 10 °C/min, holding them at 900 °C for 120 min under CH_4_/N_2_ or H_2_/N_2_ atmosphere, and finally oxidizing them in CO_2_ and air for 30 and 15 min, respectively (also at 900 °C). The isothermal cyclic redox experiments were performed with similar initial steps as the TPR to dry the materials. The reduction stage was carried out isothermally at 900 °C in 5% CH_4_. The reduced materials were replenished isothermally at 900 °C in CO_2_ and then air for 30 min each.

### 2.3 Chemical Looping Syngas Production in the Fluidized Bed

C_2_F performance in redox cycles was demonstrated in a fluidized bed (shown in supplementary Figure S1). The reactor consisted of an alumina tube (i.d. 20 mm) with a distributor which located the fluidized bed in the heated region. The bed was heated externally by a tubular furnace, and the bed temperature was controlled by a K-type thermocouple and feedback controller. Gases were fed to the bottom of the reactor via mass flow controllers and solenoid valves. The composition of the outlet gas was measured using gas analyzers (ABB EL3020) equipped with a nondispersive infrared (NDIR) cell for CO, CO_2_, and CH_4_, a paramagnetic cell for O_2_, and a thermal conductivity sensor for H_2_. Water was not measured directly but instead inferred by balances (details given in the Supporting Information – S10). The gases were sampled by a diaphragm pump (16 mL s^–1^) and then dried using a glass tube filled with CaCl_2_, before being sent to the gas analyzers.

A typical fluidized bed experiment involved feeding 0.8 g of C_2_F (500–850 μm, density ∼ 1500 kg/m^3^) into a preheated bed of recrystallized alumina sand (∼40 g, size 350–420 μm, Boud Minerals, grade WA 46) initially fluidized by N_2_. Reacting gases were supplied from gas cylinders (BOC) of 5% CH_4_/N_2_, 100% CO_2_, compressed air, N_2_ and 5% H_2_/N_2_. The total flow rate was ∼33 mL s^–1^ at NTP (20 °C, 1 atm), and accordingly the bed of particles was fluidized with *U*/*U*_*mf*_ ∼ 10, i.e., where *U* is the superficial velocity of the fluidizing gas, and *U*_*mf*_ is the minimum fluidization velocity. Redox cycle experiments were performed for ∼35 cycles; unless stated, each cycle consisted of reduction with 5% CH_4_/N_2_ for 60 min, followed by regeneration (i.e., oxidation) using 20% CO_2_/N_2_ for 15 min and then air for a further 15 min. Between stages, the bed was purged with N_2_ for 4 min, of which only 2 min are shown in the following results; for the remaining 2 min, the reacting gas mixture was diverted through the gas analyzer to measure the inlet composition fed during the reaction.

### 2.4 Material Characterization

The fresh and after-cycled materials were characterized using X-ray diffraction (XRD) analysis (Siemens D500 X-ray diffractometer with Cu K_α_ radiation). The materials were prepared on an aluminum mount; thus, a blank experiment was also performed without samples on the mount. The XRD was operated at 35 kV and 20 mA, and a scan range between 5° and 90° in 2 θ and a step size of 0.02° was used. Phases were identified by comparison with reference patterns from the Inorganic Crystal Structure Database (ICSD). Scanning electron microscopy (SEM) images were obtained using a TESCAN MIRA3 FEG-SEM at 15 kV. The SEM was equipped with an energy-dispersive X-ray (EDS) detector (Oxford Instruments Aztec Energy X-maxN 80). For SEM-EDS, the samples were placed on carbon adhesive discs (Agar Scientific) and sputtered with a 10 nm layer of platinum (Quorum Technologies 150T ES).

## 3 Results

### 3.1 Temperature-Programmed Reduction (TPR) and Cyclic Reduction in CH_4_ and Isothermal Oxidation in the TGA

**Figure 2:**
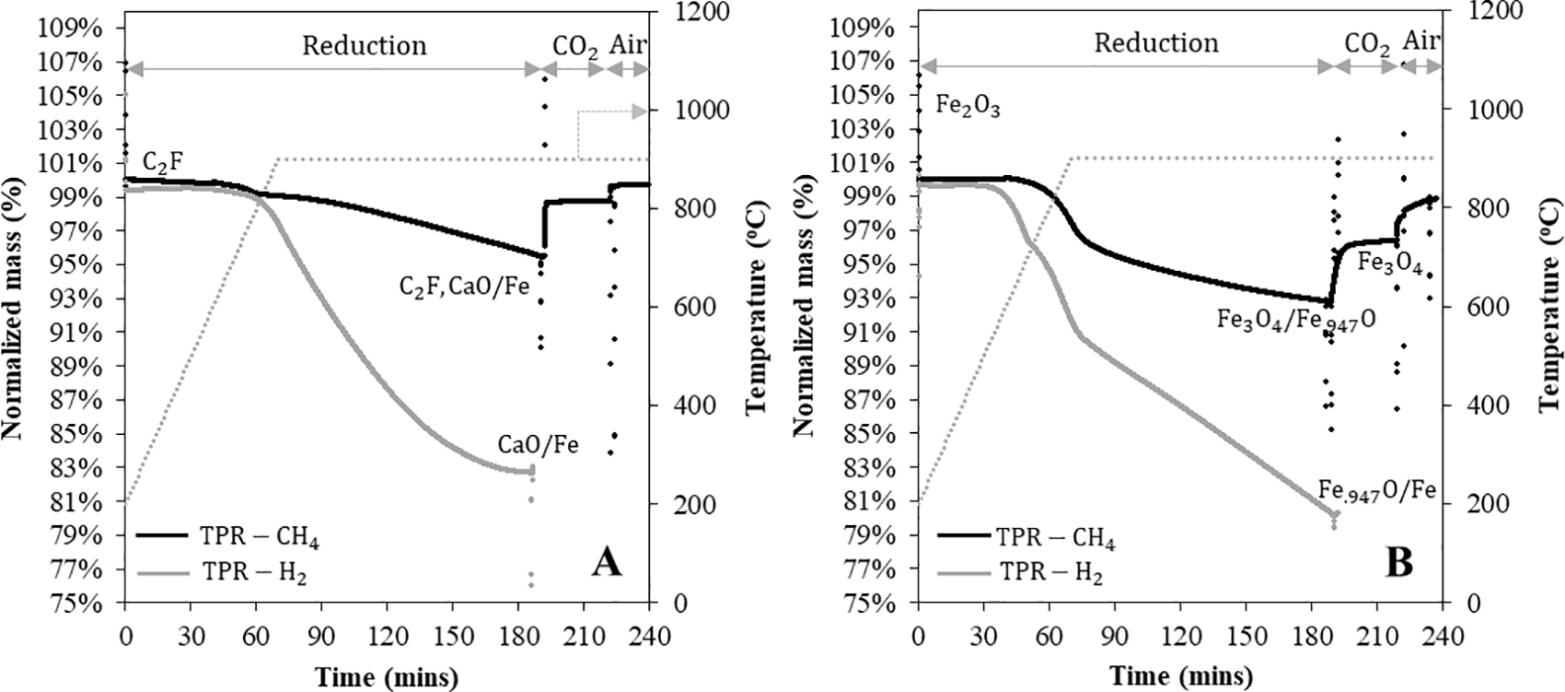
Temperature-programmed reduction (TPR) in CH_4_ (**—** (black)) or H_2_ (— (gray)) in the TGA of [**A**] C_2_F and [**B**] Fe_2_O_3_: the samples were heated from 200 to 900 °C at a heating rate of 10 °C/min and held for 120 min at 900 °C and for TPR in CH_4_ followed by CO_2_ (30 min) and then air (15 min) oxidation.

The TPR of C_2_F and Fe_2_O_3_ from 200 to 900 °C, followed by isothermal reduction at 900 °C, is presented in Figure 2. After reduction in CH_4_, the samples were oxidized using CO_2_ and then air. The corresponding differential (i.e., DTG) curves are given in Figure S2 in the Supporting Information. In Figure 2, neither the fresh C_2_F nor Fe_2_O_3_ was fully reduced by CH_4_, losing only 0.045 and 0.07 g/g, respectively, compared with 0.177 and 0.3 g/g when they are fully reduced into metallic Fe.

**Figure 3:**
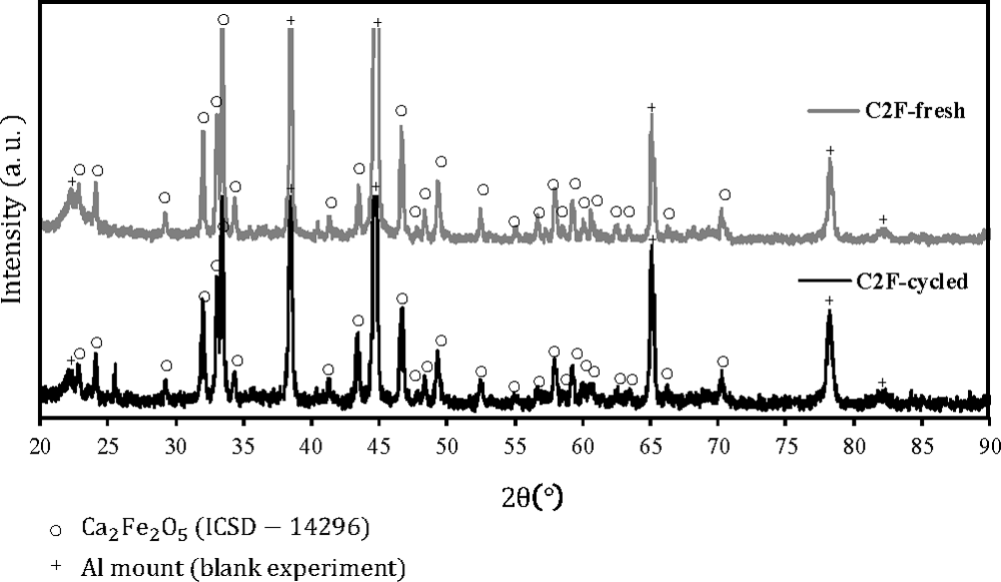
XRD patterns of fresh C_2_F (—) and after 37 cycles (**—** (bold)) in the fluidized bed at 900 °C. The reference pattern was obtained from ICSD – 14296 for Ca_2_Fe_2_O_5_ (C_2_F), labeled “○”.

From the XRD pattern for this sample in Figure 3, the material appeared to be phase pure C_2_F. C_2_F should reduce in a single step;^24^ however, the reduction in methane appeared to undergo two steps, with a very small change in mass during the temperature ramp, but with the bulk of the reduction taking place after reaching 900 °C. The first small mass loss under CH_4_ is likely to be impurity phases that are below the detection limit of the XRD, but which appear to contribute significantly to the reduction under CH_4_, simply because C_2_F in this fresh sample is very unreactive. The fact that some air was needed for oxidation is also indicative of impurity phases. This can be contrasted to the TPR under hydrogen, which was much faster, so it goes to completion in the time allowed, showing only one step, presumably as the reduction of any small impurity phase is not that significant and is masked by the much larger reduction of C_2_F. The fresh C_2_F only started to react with CH_4_ at ∼900 °C. In contrast, as shown in Figure 2, Fe_2_O_3_ reacted at ∼700 °C and showed multiple reactions, indicative of the reduction through different iron oxides.

**Figure 4:**
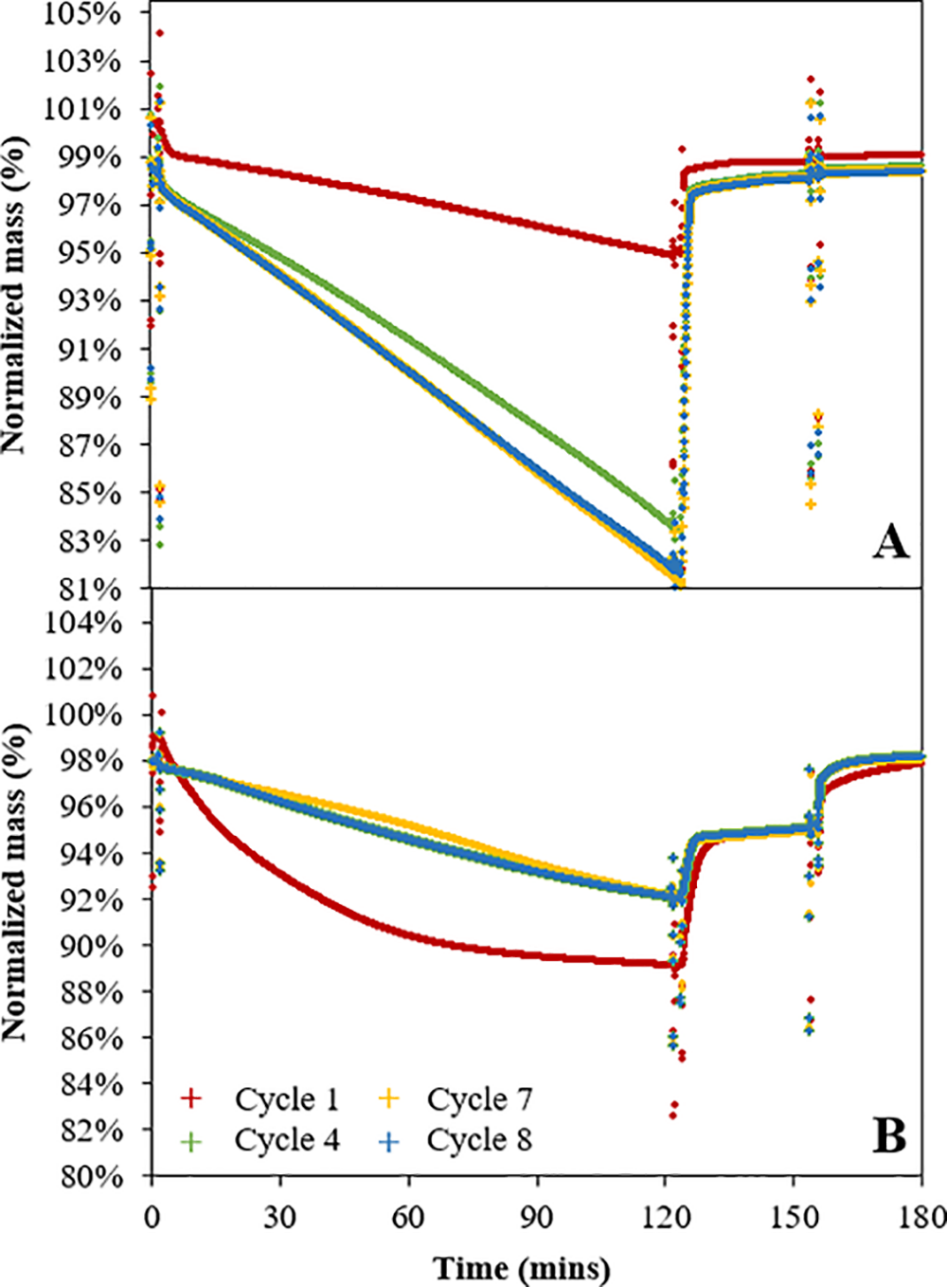
Isothermal redox cycling experiments in the TGA at 900 °C of [**A**] C_2_F and [**B**] Fe_2_O_3_: (i) reduction in CH_4_ for 120 min, (ii) oxidation in CO_2_ for 30 min, and (iii) oxidation in air for 30 min. %Mass was normalized to the material’s mass after drying in air at 900 °C for 30 min.

Figure 4 shows the results for isothermal reduction–oxidation (redox) cycling of the material at 900 °C in the TGA. In the first cycle, there was a small gap between the initial mass and mass at the end of the cycle; C_2_F and Fe_2_O_3_ recovered 98% and 99% of their mass, respectively. This is likely caused by a small amount of carbonation or moisture in the fresh sample which was not completely removed during the drying step. In subsequent cycles, the final mass after air oxidation was approximately constant. The extent of reduction of C_2_F in CH_4_ improved over cycles, reaching its maximum mass loss after the fifth cycle, after which it remained relatively stable. In comparison, the mass loss of Fe_2_O_3_ during reduction was relatively stable over 8 cycles, but at a low value ∼0.06 g/g-material, i.e., 20% of its theoretical oxygen transfer. Unsupported Fe_2_O_3_ by itself will experience severe sintering causing deactivation of the material if completely reduced.^24,31^ The only reason this appears not to happen in Figure 4B is that very little reduction occurs as the iron oxide is very unreactive toward the CH_4_.

Theoretically, fully reduced C_2_F (a mixture of metallic Fe and CaO) should have been able to be fully replenished back into C_2_F using CO_2_ or steam. During the isothermal redox cycles, the reduced form of C_2_F was capable of being largely fully regenerated using only CO_2_; very little oxidation was seen when the oxidant was switched to air. This can be seen from the % mass difference between oxidation in CO_2_ and air, i.e., 97.8 wt % vs 98.7 wt % in Figure 4A. If full segregation between Fe_2_O_3_ and CaO occurred and the oxidation in CO_2_ replenished the metallic Fe into Fe_3_O_4_, instead of incorporating it back into C_2_F, the gap should have been ∼2 wt %. The 0.94 wt % mass difference could have been caused by either unstable TGA balance or kinetic limitation, i.e., a longer CO_2_ oxidation may be needed to fully regenerate the reduced C_2_F. On the other hand, for Fe_2_O_3_, the equilibrium only allows the sample to readily oxidize to magnetite using CO_2_ as demonstrated in Figure 4B, and air is needed to complete the oxidation.

### 3.2 Chemical Looping Syngas Production in the Fluidized Bed

**Figure 5:**
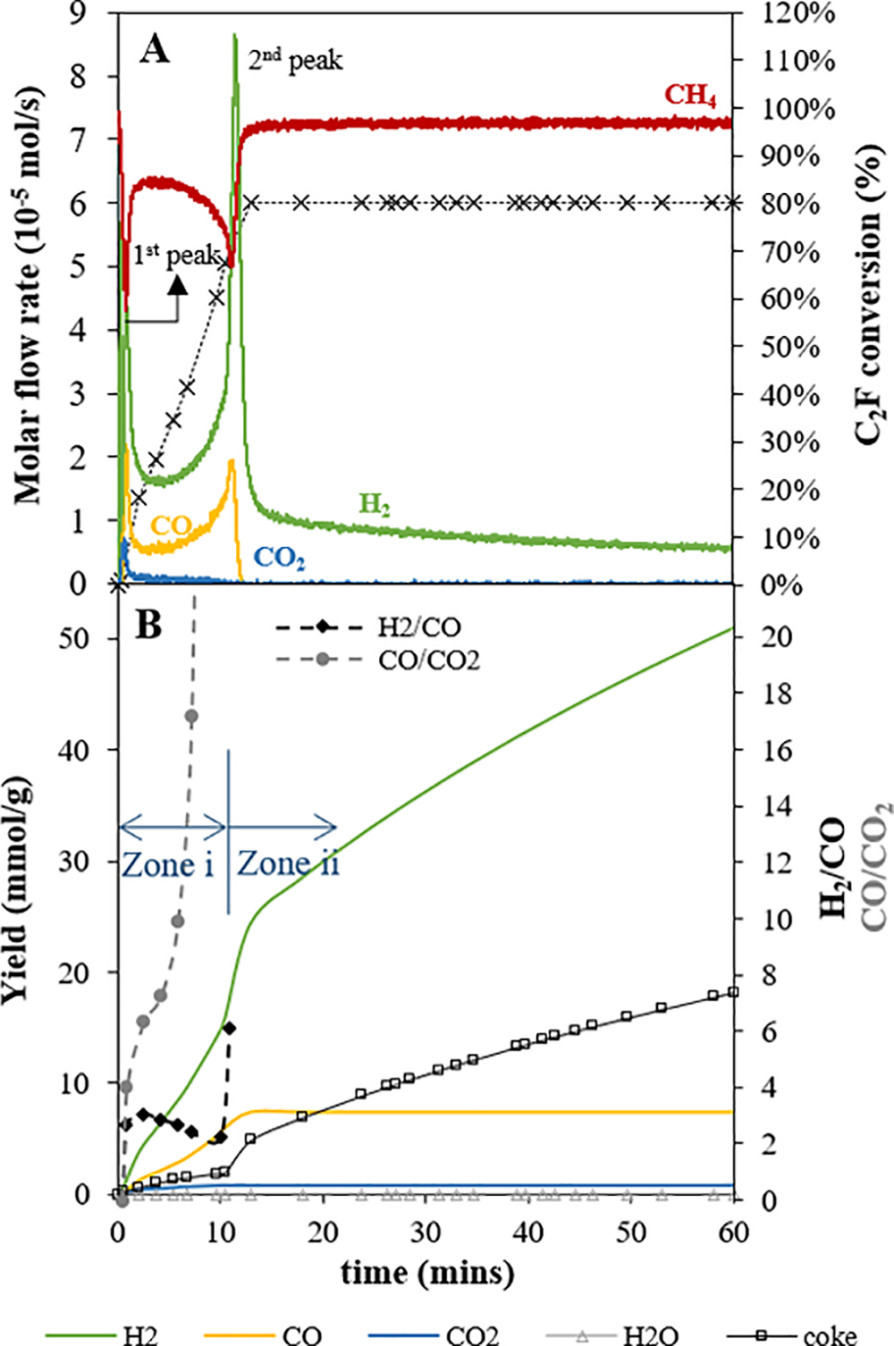
Results from a typical cycle (7th cycle) in the C_2_F cycling experiments in the fluidized bed at 900 °C: [**A**] off-gas profile expressed as the molar flow rate and conversion of C_2_F (— (red) CH_4_, — (green) H_2_, — (yellow) CO, — (blue) CO_2_) and [**B**] corresponding cumulative yields and syngas ratios, i.e., [H_2_]/[CO] and [CO]/[CO_2_].

The reducibility of C_2_F in CH_4_ and its ability to perform multiple chemical looping partial oxidation cycles were also examined in the fluidized bed; Figure 5 shows a typical cycle (in this case cycle 7 of 37) when 5% CH_4_/N_2_ was fed to the fluidized bed. Following this, two stages of oxidation were carried out (1) in an atmosphere of 20% CO_2_/N_2_ and (2) in air to completely replenish the reduced materials. In Figure 5, three distinct behaviors can be seen: (i) the initial rapid but short-lived methane consumption, followed by (ii) a slow reaction, then by (iii) an acceleration in rate (shown in the graph by a dip in the methane flow from the reactor) which peaks. Over this period the same behavior is reflected in the CO and H_2_ production rates, showing significant partial oxidation of the methane. It would appear that if there was coking it was not detrimental to the oxygen transfer. After the second peak in methane consumption, oxygen transfer fell off, but methane continued to be consumed and hydrogen produced, albeit at a slower rate, with the dominant reaction being methane cracking,  ⇋ C_(s)_ + .

**Figure 6:**
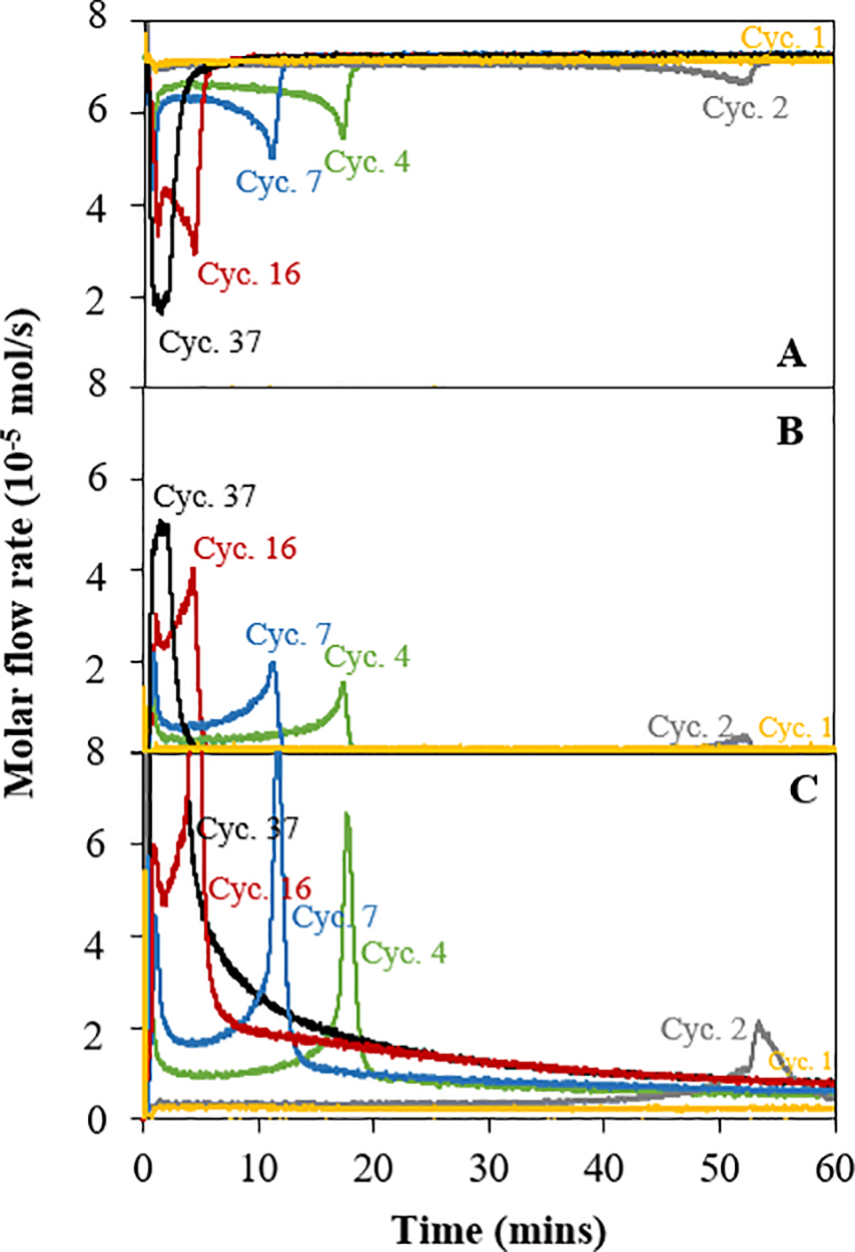
Evolution of the profile of off gases during the reduction in the CH_4_ stage over 37 cycles in the fluidized bed at 900 °C: [**A**] CH_4_ [**B**] CO, and [**C**] H_2_.

**Figure 7:**
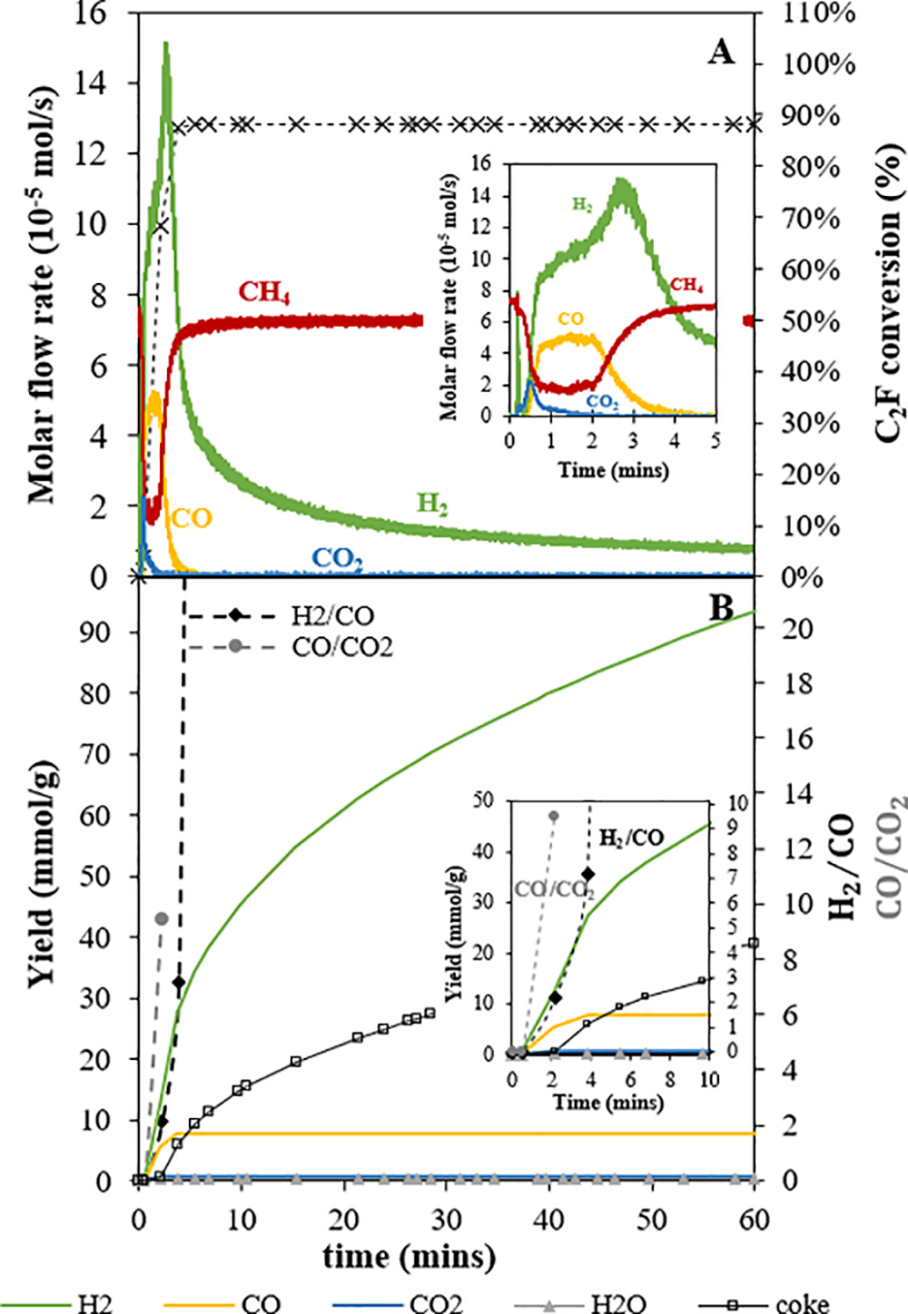
Reaction profile from the final cycle (37th) during reduction in CH_4_ using C_2_F in the fluidized bed at 900 °C: [**A**] molar flow rate of outlet gases and C_2_F conversion and [**B**] cumulative syngas yield and syngas ratio, i.e., [H_2_]/[CO] and [CO]/[CO_2_]. The inset shows the initial behavior.

Figure 6 shows that C_2_F evolved into a more active oxygen material transfer with cycling. In early cycles (<3rd cycle), the CH_4_ reduction had poor kinetics, indicated by a similar CH_4_ molar flow at the inlet and outlet of the fluidized bed, and little production of CO, CO_2_, or H_2_O. In fact, the fresh material shows almost no initial activity toward methane, and there is a long induction time before seeing any reaction. After cycle 3, there was still not only an induction time but also an initial (<5 min of exposure) rapid methane consumption, followed by a slower reaction which then accelerated between 10 and 20 min. As the material was cycled and became more active, the second peak shifts to early times (as shown in Figure 6), leading to the profile in Figure 7 in cycle 37.

**Figure 8:**
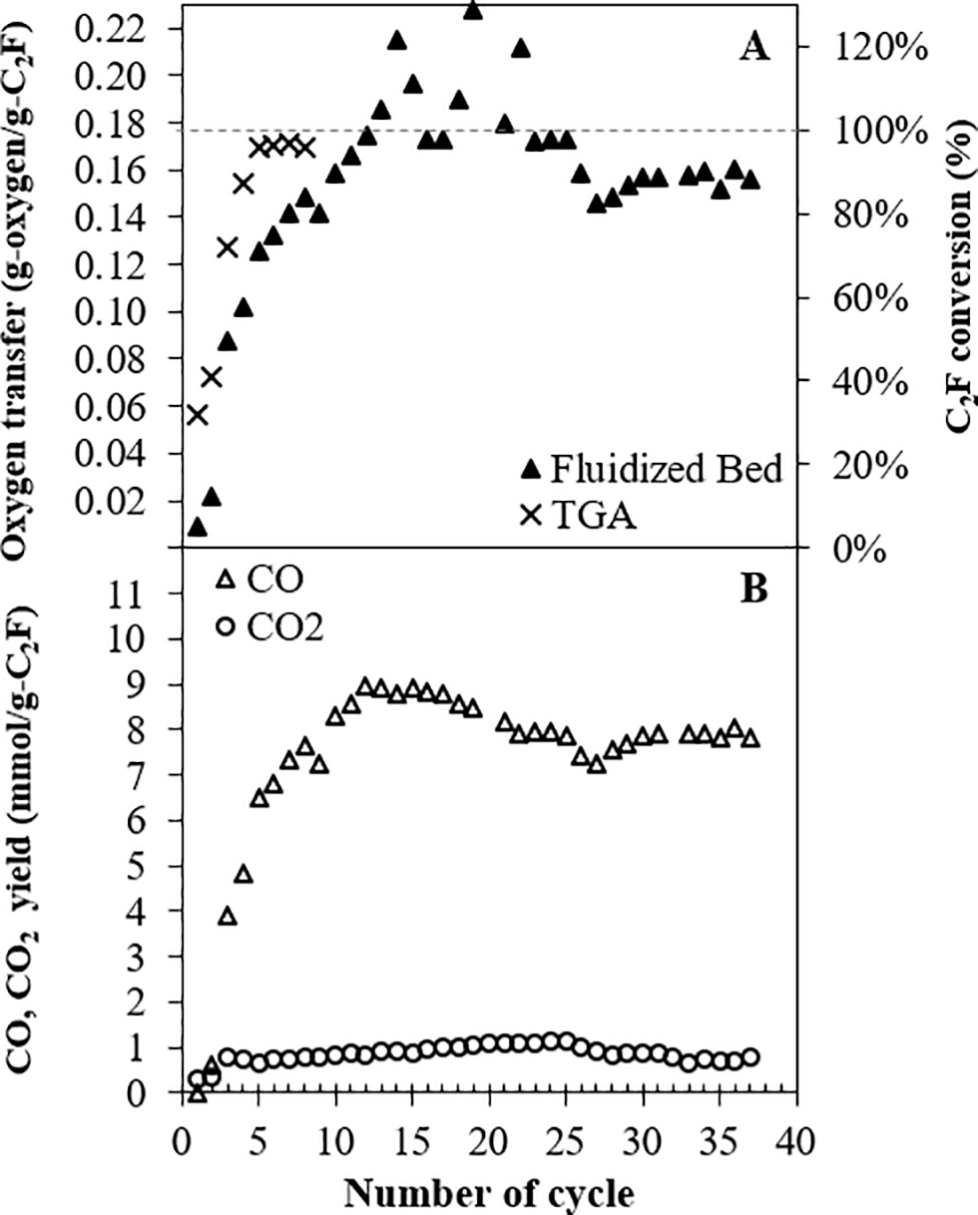
Yields and capacities measured during isothermal cycling of C_2_F at 900 °C: [**A**] oxygen transfer (left axis) and corresponding C_2_F conversion for full reduction to CaO and Fe (right axis) during reduction in the TGA (×) and fluidized bed (▲) and [**B**] CO (Δ) and CO_2_ (○) yields on each cycle during the reduction in the fluidized bed. The TGA cycle consisted of (i) a 120 min reduction in CH_4_/N_2_, (iii) a 30 min oxidation in CO_2_/N_2_, and (iii) a 30 min oxidation in air. The fluidized bed cycle consisted of (i) a 60 min reduction in 5% CH_4_/N_2_, (iii) a 15 min oxidation in 20% CO_2_/N_2_, and (iii) a 15 min oxidation in air.

Figure 8 shows the amount of oxygen transferred from C_2_F during the reduction phase in each cycle in the fluidized bed; also shown for comparison are oxygen transfer capacities measured in the TGA in similar cycles. Here, the conversion is based on the oxygen balance, i.e., total yield of oxygen in CO, CO_2_, and H_2_O, divided by the total oxygen expected by reducing C_2_F completely to CaO + Fe. A very small amount of syngas was produced during the first cycle in the fluidized bed, and C_2_F gave up 0.9 wt % of its oxygen (i.e., a conversion of only ∼5%). At a higher number of cycles, C_2_F was able to almost attain its maximum oxygen transfer capacity and was relatively stable in subsequent cycles.

The results from the fluidized bed are comparable to those in the TGA. In both experiments, the oxygen transfer of C_2_F improved as the number of cycles increased. The fluidized bed occasionally appeared to give conversions greater than 100%; however, this indicates some experimental error in these particular cycles. Agreement between the TGA and fluidized bed indicates errors are low, and at worst, the error in conversion is only 20%. Conversion is based on the oxygen transfer capacity and is calculated from CO, CO_2_, and H_2_O yields, where the H_2_O yield is itself inferred by balance. Thus, conversion can be sensitive to accumulated errors. For comparison, yields for single components would typically be accurate to within 5%. It should be noted that the times for each reaction phase had to be extended in the TGA owing to the much slower reaction when compared with the fluidized bed. This noticeable difference in rate can be attributed to the effects of mass transfer which are less limiting in the fluidized bed.

When C_2_F transfers its lattice oxygen to CH_4_ during reduction in the fluidized bed (Figure 5), the low value of *P*_O_2__ for C_2_F_(s)_ ⇋ 2CaO_(s)_ + 2Fe_(s)_ +  should ensure gaseous products are mainly CO and H_2_, as demonstrated in Figure 5. Figure 8B shows the CO yield during the reduction phase alone was significant, but the yield of CO_2_ was almost much lower. C_2_F selectivity toward syngas production is therefore relatively high. Given the stable oxygen transfer shown in Figure 8A, it is unsurprising that Figure 8B shows a relatively stable yield of CO through the eighth to 37th cycles.

**Figure 9:**
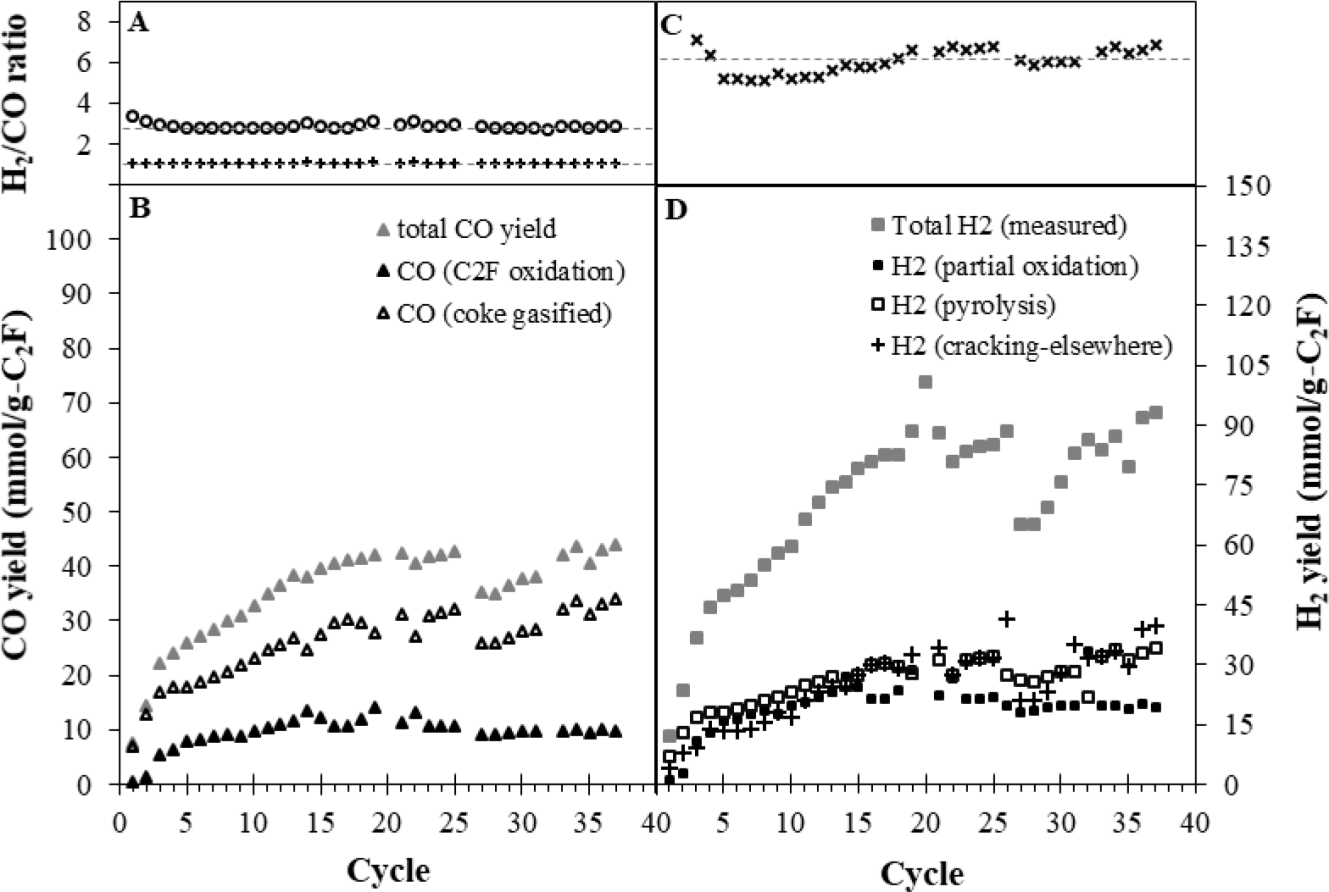
[**A**] [H_2_]/[CO] ratio overall in the fluidized bed based on total syngas yield during reduction in CH_4_ and oxidation in CO_2_ (+) and hypothetical oxidation in steam (○). [**B**] (▲ (gray)) Total CO yield measured during the CO_2_ oxidation phase alone; (▲) CO yield expected from the oxidation of reduced C_2_F; (Δ) is the estimated CO yield from gasifying coke formed. [**C**] [H_2_]/[CO] ratio overall based on total syngas yield during reduction in CH_4_ alone (×). [**D**] H_2_ yield for reduction in 5% CH_4_/N_2_ for 1 h in the fluidized bed for 37 cycles: Total measured H_2_ yield (■ (gray)); H_2_ from partial oxidation (■) is the stoichiometric yield based on the CO yield; H_2_ from pyrolysis (□) is estimated from the excess CO yield produced during the CO_2_ oxidation; the remainder can be attributed to H_2_ from cracking elsewhere (+).

Oxygen transfer capacities are based on the oxygen balance and thus are not complicated by coking. Hydrogen production and CH_4_ consumption, however, are affected by coke formation. Throughout the reduction phase in CH_4_, the H_2_ produced was larger than the theoretical amount predicted from the CO yield via  + C_2_F_(s)_ ⇋ 3CO_(g)_ +  + 2CaO_(s)_ + 2Fe_(s)_. This excess H_2_ likely arose from pyrolysis, i.e.,  ⇋ C_(s)_ +  (i) on the C_2_F material surface or (ii) elsewhere in the fluidized bed considering the high temperature of the bed material. A blank experiment cycling was performed in a fluidized bed filled with alumina sand alone. The outlet gas profile (given in Figure S4 in the Supporting Information) showed negligible CH_4_ reacted, i.e., methane cracking within the system was not significant in the absence of C_2_F. The excess H_2_ produced was estimated by subtracting total H_2_ yield from theoretical H_2_ yield from CH_4_ partial oxidation and used to determine the cumulative coke yield which is shown in Figure 9.

Considering the seventh cycle in Figure 5, in the time leading up to the second maximum in CH_4_ consumption (*t* = ∼10 min, when C_2_F conversion ∼ 67%) around 2 mmol/g of coke was deposited (estimated from the ∼4 mmol/g of excess H_2_). During this period, the [H_2_]/[CO] ratio (Figure 5B) was relatively stable at ∼2, indicating partial oxidation of CH_4_ dominated the reaction during this period. During the second peak in methane consumption (*t* = 10–13 min), the conversion of C_2_F reached its maximum, i.e., 80%, and as oxygen transfer finished (i.e., CO production fell to zero at *t* = 13 min), the [H_2_]/[CO] ratio rapidly rose. During this period, the coke yield increased to 5 mmol/g (corresponding to an excess H_2_ yield from pyrolysis of ∼10 mmol/g), while H_2_ produced from the partial oxidation of CH_4_ was ∼15 mmol/g. The H_2_ formation then continued without oxygen transfer (zone **ii** in Figure 5) until the end of this phase of the cycle, reaching ∼50 mmol/g and giving ∼35 mmol/g synthesized from the methane pyrolysis alone. This means that a total of 18 mmol/g of coke were produced in the 1 h of reaction, mostly after the oxygen transfer had finished. To directly measure coke formed, in the 26th cycle, the oxidation was completed under air only; the amount of CO and CO_2_ generated was 13.1 and 5.7 mmol/g-C_2_F, respectively, which corresponds to 18.7 mmol/g of coke produced during the CH_4_ reduction phase at the 26th cycle.

The coke produced during the reduction can also be inferred from an excess CO yield during the following CO_2_ oxidation. Figure 9B gives the CO yields from the CO_2_ oxidation stage in the fluidized bed experiments. Taking the amount of CO generated in this CO_2_ oxidation phase in the seventh cycle as an example, i.e., 28.5 mmol-CO/g, this exceeds the maximum theoretical yield from C_2_F regeneration (11 mmol-CO/g, if C_2_F is fully reduced). C_2_F only reached 80% conversion in the seventh cycle which is associated with 8.9 mmol-CO/g-C_2_F. The excess of ∼20 mmol-CO/g arises from C_(s)_ +  ↔ 2CO_(g)_ and means ∼10 mmol/g of coke must have been deposited onto the C_2_F surface in the reduction phase (8 mmol/g less than the estimate based on excess H_2_ yield). There was no CO or CO_2_ released during the air stage, indicating all the coke was removed during CO_2_ oxidation.

The last cycle shown in Figure 7 shows only one peak at the beginning. Similar to the seventh cycle, minimal coke was generated when the oxygen transfer rate was high, inferred from the CO production. A significant difference was the total H_2_ yield produced between the early (seventh) and the last cycle (37th). During the initial period when there was oxygen transfer (*t* < ∼10 min at the seventh cycle and ∼2 min at the 37th cycle), the H_2_ yield was similar, ∼15 mmol/g for both cycles (see Figures 5B and 7B). However, at the end of reaction, the total H_2_ yield was 50 vs 90 mmol/g, giving an excess H_2_ yield of ∼35 and ∼75 mmol/g, for the seventh and 37th cycles, respectively. On the other hand, according to the CO excess yield during the CO_2_ oxidation phase, the excess H_2_ should be only ∼20 and ∼34 mmol/g, respectively. This suggests that the discrepancy in the H_2_ produced became more significant in later cycles.

Coke deposition was also observed during TPR experiments of the after-cycled C_2_F. After ∼35 cycles in the fluidized bed, some materials were retrieved, and a TPR experiment in CH_4_ was performed. In contrast with the TPR for fresh material, mass increased at the end of reduction, suggesting coke formation (see Figure S5 in the Supporting Information).

Methane cracking was observed elsewhere within the fluidized bed system, with black solid carbon deposited in the quartz sampling tube and also onto the freeboard of the fluidized bed, and observed to be more significant toward the end of cycling. This correlated with the much higher discrepancy in excess H_2_ yield, estimated from the CO on oxidation or H_2_ during reduction; e.g., ∼27% discrepancy at the seventh cycle and ∼43% at the 37th cycle, which would correspond to an estimated ∼8 and ∼22 mmol/g of coke unaccounted for. As long as methane cracking occurs after the zone where the gases are sampled, it will not have any effect on the measurements. However, any cracking prior to or near the sampling point will result in excess H_2_ being produced. Figure 9D gives a breakdown of the amounts of hydrogen produced on each cycle. Here, the H_2_ generated from methane cracking elsewhere was estimated from the aforementioned discrepancy.

Figure 9B shows that oxidizing the reduced C_2_F using CO_2_ produced additional CO. In a case where steam is utilized instead of CO_2_, additional H_2_ could be produced via (i) 2CaO_(s)_ + 2Fe_(s)_ + 3H_2_O_(g)_ ⇋  +  and (ii) C_(s)_ + H_2_O_(g)_ ⇋ CO_(g)_ + . Considering the similar oxygen potential of CO_2_ and steam, i.e.,  ∼ 2.5 compared with  ∼ 3.3 at 900 °C for the equilibrium at which metallic Fe and CaO are replenished to C_2_F, in this current work, the material was only regenerated in CO_2_ and not with steam to avoid the complications of feeding steam.

The average [H_2_]/[CO] ratio can be varied with the duration of the reduction phase, e.g., partial oxidation alone vs both partial oxidation and methane pyrolysis. Figure 9A shows [H_2_]/[CO] obtained from the total syngas yield within the cycle overall. The total syngas generated during reduction alone yielded an average [H_2_]/[CO] ratio of ∼6, whereas if it was combined with the additional CO or H_2_ generated during oxidation in CO_2_ or steam, [H_2_]/[CO] would be around 1 or 3, respectively.

### 3.3 The Reduced C_2_F: An Active Methane Pyrolysis Catalyst

**Figure 10:**
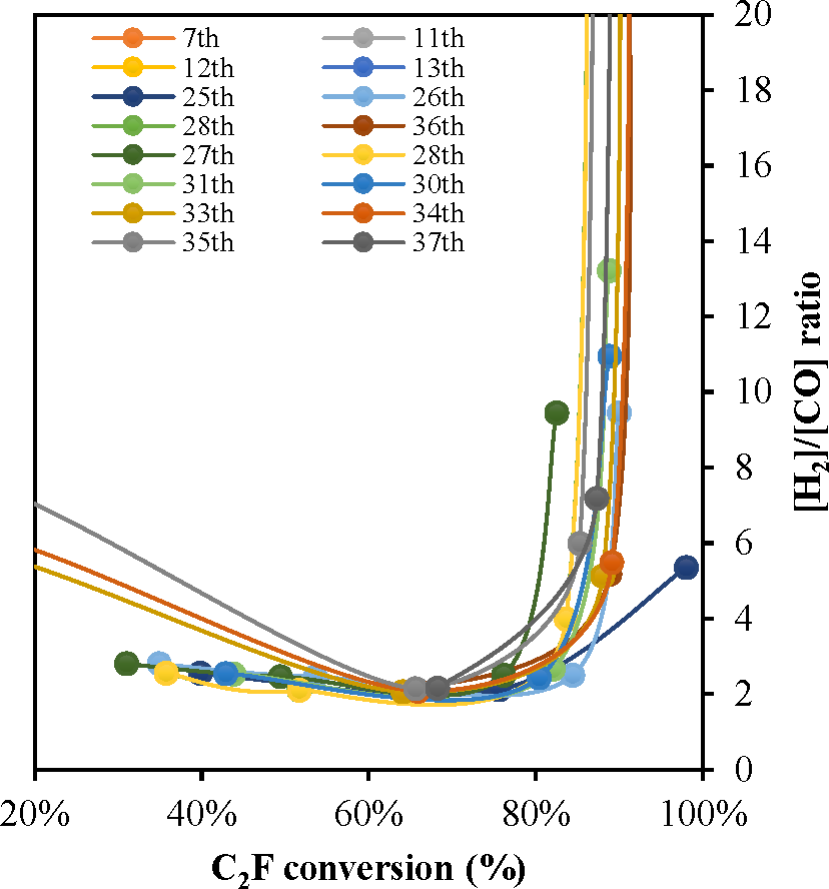
C_2_F conversion (based on oxygen transferred) vs the [H_2_]/[CO] ratio measured at the outlet of the reactor, during reduction in the fluidized bed cycle.

At the end of the cycle, methane pyrolysis dominates (zone ii in Figure 5), implying the reduced C_2_F is an active methane pyrolysis catalyst. Prior to this, there was an induction period (which shortened as the cycles proceeded (e.g., Figure 6)), leading to an accelerating rate, and a second peak in methane consumption (i.e., zone i in Figure 5). The second peak in methane consumption coincided with a rapid rise of [H_2_]/[CO], when the C_2_F conversion reached > ∼80% as shown in Figure 10. The catalytic activity in zone ii and the induction period and accelerating rate suggest that the Fe produced as the material reduces is important for methane conversion in both stages.

**Figure 11:**
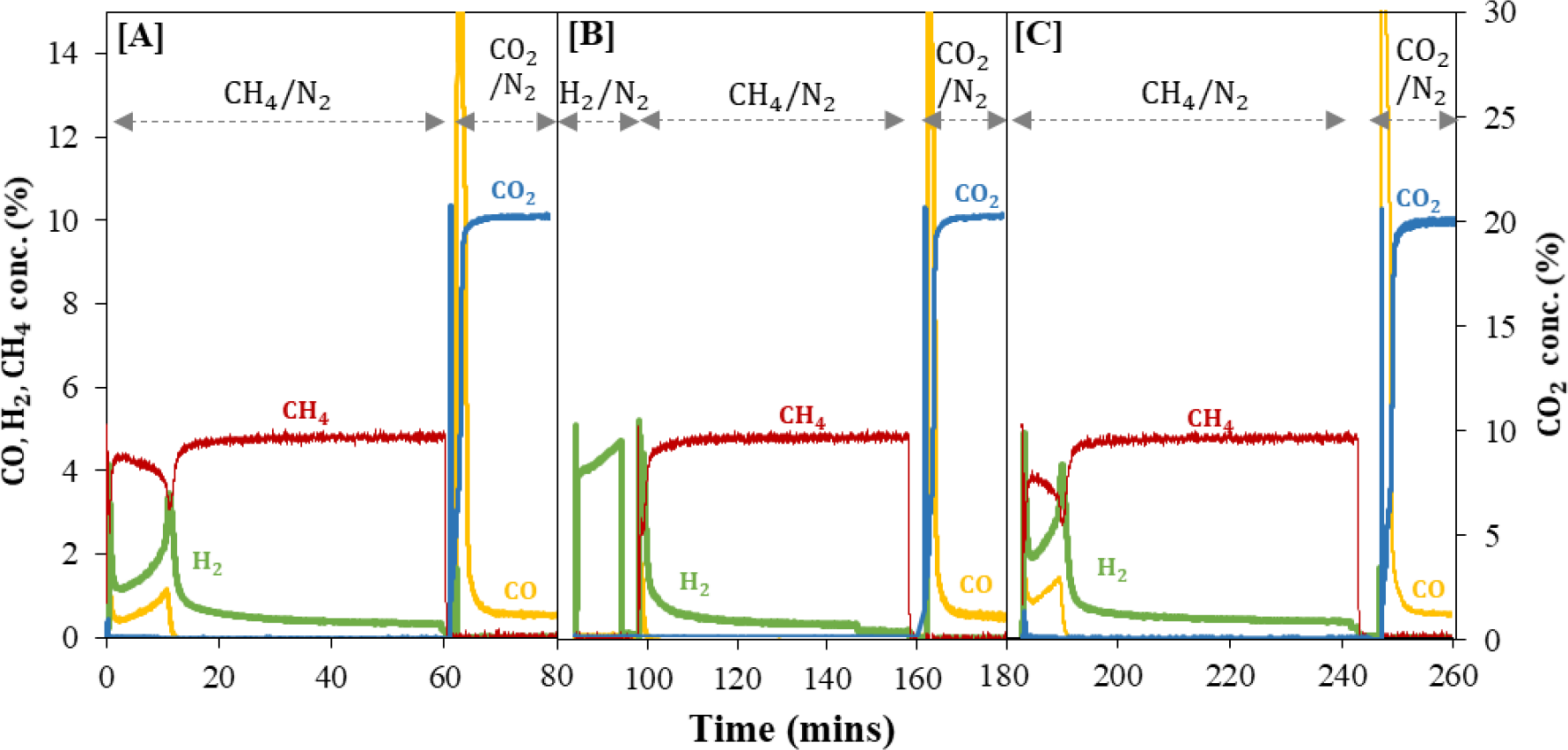
Off-gas concentration profile in an isothermal redox experiment at 900 °C: [**A**] cycle 9, 0.8 g of retrieved C_2_F was reacted in 5% CH_4_/N_2_ for 60 min and oxidation in 20% CO_2_/N_2_ for 20 min, [**B**] cycle 10, 5% H_2_/N_2_ for 10 min followed by 5% CH_4_/N_2_ for 60 min and oxidation in 20% CO_2_/N_2_ for 20 min, and [**C**] cycle 11, 5% CH_4_/N_2_ for 60 min and oxidation in 20% CO_2_/N_2_ for 20 min.

The ability of reduced C_2_F to catalyze methane pyrolysis was evaluated by initially activating a 2 g batch of the fresh C_2_F via the typical CH_4_ reduction cycle for 8 cycles in the fluidized bed. Next experiments on 0.8 g of this activated C_2_F (using fresh alumina sand and a clean reactor to avoid any confounding effect of contamination) first performed a typical cycle (i.e., cycle 9 in Figure 11A), followed by a cycle (cycle 10 in Figure 11B) in which the material was exposed to 5%H_2_/N_2_ at 900 °C for 10 min to reach a conversion of ∼80% (see Figure S6 in the Supporting Information) before then exposing the sample to 5% CH_4_/N_2_. Figure 11B shows methane consumption instantly after methane was fed, i.e., there was no induction period. In addition to the conversion of the solid by H_2_ in Figure 11B, reaction with methane produced 2 mmol/g of CO giving a further 18% conversion of C_2_F, i.e., 98% in total. Thus, the products of C_2_F reduction appear to accelerate both methane pyrolysis and also the oxidation of methane by the oxygen contained in the C_2_F. Following this, C_2_F was regenerated in 20% CO_2_/N_2_ for 20 min and then exposed to 5% CH_4_/N_2_, as shown in Figure 11C, in which the induction period between the first and second peak has reappeared.

## 4 Discussion

In general, the material shows an initial–but short duration–high reactivity toward methane in which first only CO_2_ is produced, followed shortly after, ∼15 s, by the production of H_2_ and CO (see Figure 5). The fact that CO_2_ (and presumably H_2_O, which was not measured) was produced alone in the early phase of the cycle might either be an indication of a small amount of phase segregation of C_2_F or the presence of highly active oxygen species on the surface. Whatever the source, once this small amount of active oxygen was depleted, i.e., when C_2_F was donating its lattice oxygen, mostly CO was generated, as would be expected from the equilibrium.

In contrast, H_2_ appeared almost immediately, and its profile followed the CH_4_ consumption profile (see Figure 5 in zone i). This might indicate methane dehydrogenation occurred by initially depositing carbon onto the material surface, releasing H_2_ which is then subsequently oxidized to form CO and/or CO_2_. Following this initial peak in material activity, the rate of consumption of methane then fell, before accelerating again to produce a second peak in methane consumption. During this second peak in methane consumption, the H_2_ production reached its maximum slightly after the rates of consumption of CH_4_ and production of CO reached their maxima. During this second peak in CH_4_ consumption, the reaction was a combination of CH_4_ partial oxidation and pyrolysis.

Coke gradually started to appear just after C_2_F was exposed to the CH_4_, but its rate of formation was low during the initial partial oxidation phase (zone i in Figure 5A). This can also be seen from the [H_2_]/[CO] ratio in Figure 5B, which should be 2 if there is only methane partial oxidation. Initially, H_2_ was produced at a relatively low rate, and coke formation was minimal, until ∼40% solid conversion. Between ∼40% and ∼60–70% conversion the rate of H_2_ production and CH_4_ consumption accelerated, and there was also an increase in the oxygen transfer rate from C_2_F. Presumably, there was still sufficient lattice oxygen, to minimize carbon buildup, with [H_2_]/[CO] only slightly greater than 2. Initially, the rate of CO + H_2_ production is low, indicating methane can directly react with C_2_F, albeit with difficulty. However, the acceleration in rate when there is significant Fe^0^ produced suggests it plays an important role in the reaction. After ∼80% C_2_F conversion, coke deposition rapidly accelerated. Thus, when reduced to Fe^0^ and CaO, C_2_F became active as a pyrolysis catalyst, but initially the oxygen transfer rate from C_2_F was able to keep up with the rapid coke formation, thus producing CO. However, once C_2_F had been sufficiently converted, the coke deposition rate exceeded the oxygen transfer rate, and rapid coke formation occurred (zone ii in Figure 5.) In early cycles (after being activated), C_2_F was able to transfer almost all its oxygen before this happened; however, after ∼25 cycles, lattice oxygen release stopped, and rapid coke formation occurred before full conversion. In the final phase of the reaction, there is no oxygen transfer, i.e., no CO, CO_2_, or steam was generated, and only methane pyrolysis to carbon and H_2_ occurred.

A similar mechanism for the Fe_2_O_3_/NiO oxygen carrier system was suggested, in which Fe_2_O_3_ was able to transfer its lattice oxygen at a sufficient rate to the reduced Ni that the buildup of coke could be prevented, allowing the Ni to stay active while producing H_2_ from methane.^38^ A deep reduction of iron containing oxides will result in metallic Fe; metallic Fe is a known catalyst for the pyrolysis of methane.^4,6,15,34^ The rapid increase in H_2_ production was also found from a deeply reduced Fe_2_O_3_/Al_2_O_3_^6,15^ and also the perovskite La_0.8_Sr_0.2_FeO_3−δ_.^30^ Miller et al. also observed catalytic methane pyrolysis during deep reduction of CaFe_2_O_4_ in a fixed bed.^1^ CH_4_ partial oxidation requires the methane to be adsorbed on the surface, break down, and remove oxygen from the lattice. When the oxygen contained in C_2_F had been mostly removed and the partial oxidation had ended, methane pyrolysis was the dominant reaction, depositing carbon. The rate of pyrolysis fell with time, perhaps as the carbon buildup limited access of the methane to the iron surface.

The importance of metallic iron in the reaction with methane can also be seen when C_2_F was reduced under H_2_/N_2_ before it was exposed to CH_4_. Figure 11 shows that the prereduced material containing metallic iron was immediately able to consume methane with no induction period, initially partially oxidizing the methane and then pyrolyzing the methane once the material ran out of lattice oxygen. The prereduced material was also able to react with methane at temperatures as low as 700 °C. In further cycles, using first H_2_ and then CH_4_ (as in Figure 11B) at 700 and 800 °C (see Figures S7 and S8 in the Supporting Information), Figure S8 shows that the total H_2_ yield was similar at all temperatures. This implies methane was able to break down on the surface at all temperatures. In contrast, without a catalyst, and in the gas phase, methane pyrolysis occurs at temperatures above 1100–1200 °C,^5^ and little methane decomposition was seen in blank experiments. Some partial oxidation was seen at temperatures as low as 700 °C, indicated by the CO produced from the CH_4_. Noncatalytic partial oxidation with gas phase oxygen occurs at a temperature > 1000 °C, but a lower temperature can be used over a catalyst.^10,39^ The yield of CO on reduction (i.e., from methane partial oxidation) decreased on increasing the temperature to 900 °C. The lower temperature experiments produced more CO, simply as a consequence of the material not being as deeply reduced in the H_2_ prereduction. Temperature-programmed reduction in the TGA under H_2_ showed C_2_F started to react at ∼750–800 °C suggesting that the extent of prereduction at the lower temperature might have been limited (see Figures S2 and S3 in the Supporting Information). However, it is clear that even at 700 °C the prereduction did cause sufficient Fe to form to allow the methane to react. During the following CO_2_ oxidation phase, Figure S8 shows the CO yield was significantly lower at 700 °C, probably as a consequence of the deposited coke not being fully gasified and removed at 700 °C.

The behavior of the C_2_F material also evolved with the number of cycles, becoming more active. In early cycles, C_2_F transferred its oxygen lattice at a much slower rate and took longer to reach its maximum conversion. There was an initial peak of reactivity and then an induction time between the first and second CH_4_ consumption rate peaks, as shown in Figure 5. As the number of cycles increased, the induction time shortened (see Figure 6) until (>35 cycles) the two peaks merged, and there was no induction period; the C_2_F conversion reached its maximum within less than 5 min as shown in Figure 7.

**Figure 12:**
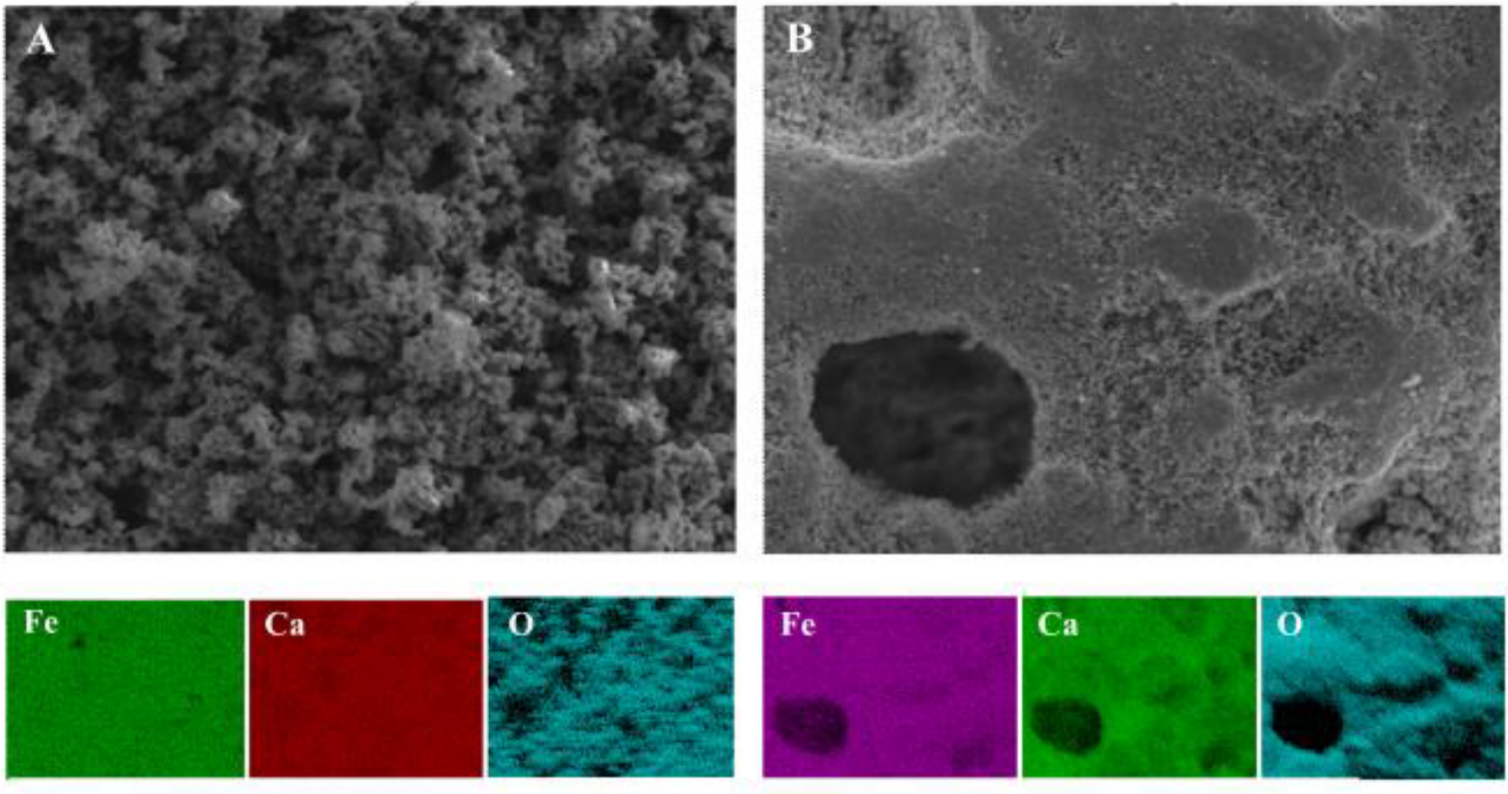
SEM and EDX results of fresh C_2_F [**A**] (Fe: 23.4%, Ca: 23.97%, O: 52.7%) and at the final cycle (cycle 37th) [**B**] (Fe: 22.9%, Ca: 15.9%, O: 61.2%).

Methane pyrolysis depositing solid carbon onto C_2_F as it reduced did not impede the transfer of oxygen. Instead, an increase in the oxygen transfer rate appeared concurrently with the catalytic methane pyrolysis. The lower oxygen transfer capacity of C_2_F at later cycles (after the 25th cycle) was likely caused by sintering of the material itself as observed in SEM (as shown in Figure 12), not because of the deposited carbon. While coking is often the main cause of catalyst deactivation and typically an issue in methane utilization processes,^40^ here it appears to be an essential step during the partial oxidation phase. The phase diagram suggests that C_2_F will reduce directly to Fe + CaO, precipitating Fe and producing dispersed iron particles. It appears that the oxygen transfer from C_2_F is determined by how fast the CH_4_ can be decomposed on the material surface, with metallic iron providing a route for methane decomposition and also acting as a reservoir storing the carbon. This is again consistent with faster oxygen transfer when more material had been reduced to metallic Fe. Once the oxygen transfer has finished, the carbon deposited can be seen as an additional source of CO, if CO_2_ is the oxidizing agent in the regeneration, since this coke is easily gasified adding to the CO produced by oxidizing the reduced C_2_F.

According to the SEM/EDX images shown in Figure 12A, fresh C_2_F contained Fe:Ca of ∼1 on its surface consistent with what is expected from C_2_F (= Ca_2_Fe_2_O_5_). Some material was retrieved after 8 cycles, when the reaction proceeds more easily and the induction time is shorter, but there was still clearly an induction time. SEM/EDX showed the Fe content was higher with Fe:Ca is ∼1.1 (see Figure S9), i.e., Fe was enriched at the surface. Toward the end of cycles, when the induction period has gone, iron seemed to segregate, leading to an enriched iron content on its surface with Fe:Ca of ∼1.5 as shown in Figure 12B.

The formation of more easily reduced iron rich phases on the surface may provide the initial iron sites for methane pyrolysis and explain the lack of an induction period and the ease with which the cycled material reacts, i.e., C_2_F was fully reduced within ∼5 min in the 37th cycle compared to ∼30 min in the fourth cycle. While the surface might be segregated, phase segregation was not observed in bulk, as shown from the XRD analysis in Figure 3, with only C_2_F peaks detected from retrieved materials in the last cycle. It should be noted while these experiments did not show bulk segregation, other cycling experiments which used a larger sample of C_2_F did show Fe_2_O_3_ peaks in XRD analysis of retrieved materials after 8 cycles (see Figures S10 and S11 in the Supporting Information). Thus, whether or not the material segregates may be a function of how it is cycled.

Segregation in the cycling experiments would also be apparent from the reoxidation profiles, since the iron can only be fully reoxidized in air. However, this is difficult to see in fluidized bed experiments since the amount of oxygen that would be consumed in the final oxidation in air is small. In the TGA however, as mass is measured directly, segregation can be measured by the extent of oxidation in CO_2_ vs that in a subsequent air oxidation. For material cycled 4 times in the TGA isothermally with methane as the fuel (details given in Figure S12 in the Supporting Information), the TPR in methane was very similar to that for fresh material (Figure 2), the reduction appeared to be dominated by a single reaction occurring at >800 °C. There was a small amount of reaction below this temperature, which might have indicated a small amount of phase impurity. The cycled material was also able to be almost completely oxidized with CO_2_, following the reduction.

Methane pyrolysis contributed more than half of the hydrogen produced during the reduction step, i.e., average value of ∼28 vs 21 mmol/g H_2_ from methane partial oxidation. Furthermore, an additional 27 mmol/g CO was produced from gasifying coke, given a total CO product of ∼38 mmol/g on average from 37 cycles. This though is an artifact of the time for which the reduced material was left exposed to the methane. Oxidation of the reduced C_2_F in CO_2_ to produce CO occurred at a very rapid rate both in the TGA (as shown in Figures 2 and 4) and in the fluidized bed with CO generated as soon as the material was exposed to CO_2_ (Figure 5). Of course some of this CO could also have come from the gasification of coke. Additionally, coke would gasify to produce H_2_ and CO, i.e., C_(s)_ + H_2_O_(g)_ ⇋  + CO_(g)_, if steam were used. This way, the ratio of syngas produced from this cycle can be readily adjusted according to the downstream requirements, e.g., gas-to-liquid of Fischer–Tropsch synthesis technology requires a [H_2_]/[CO] ratio of ∼2.^41^

The partial oxidation of methane using a C_2_F oxygen carrier is extremely endothermic (Δ*H*_298 K_^°^ = +253 kJ mol CH_4_^–1^)), and both the oxidation in CO_2_ and steam are moderately exothermic (i.e., Δ*H*_298 K_^°^ = −7 kJ mol CH_4_^–1^ and −48 kJ mol CH_4_^–1^, respectively). Overall, oxidation of the methane with C_2_F and then regeneration with CO_2_ are equivalent to dry reforming, i.e.,  +  → 2CO_(g)_ + , Δ*H*_298 K_^°^ = +247 kJ mol ^–1^, giving an overall process that has a large heat requirement. Accordingly, a fraction of the oxidation of the reduced material has to be carried out using air to balance the heat: 85% (if using steam) or 87% (if using CO_2_). The overall process is a linear combination of endothermic reforming (if CO_2_ or H_2_O is the sole oxidant) and exothermic partial oxidation (if O_2_ is the sole oxidant), with the freedom to choose the extent of each reaction and overall heat load.

On the other hand, methane pyrolysis is much less endothermic than its partial oxidation with C_2_F, with Δ*H*_298 K_^°^ = 75 kJ mol CH_4_^–1^;^42^ therefore, a combination of partial oxidation and pyrolysis of CH_4_ can potentially reduce the energy requirement in the reduction phase of the process. However, if combined with regeneration in CO_2_ and the solid carbon gasified (C_(s)_ +  ⇋ 2CO_(g)_, Δ*H*_298 K_^°^ = +172 mol ^–1^), the overall process would again be simply dry reforming of methane but with the enthalpy changes distributed differently between the different phases of the cycle. If air is used as an oxidant and some carbon burns to CO_2_ (Δ*H*_298 K_^°^ = −394 kJ mol^–1^), then the amount of carbon combusted to CO_2_ is an additional degree of freedom. Arbitrary amounts of carbon can be cracked and then oxidized to CO_2_ to generate any desired quantity of heat, effectively making the process a linear combination of dry reforming of methane and the exothermic oxidation  +  →  + , Δ*H*_298 K_^°^ = −319 kJ mol^–1^.

A tunable ratio of syngas could possibly be achieved by adjusting the oxidants used. Additionally, air oxidation can be introduced into the cycle, or oxygen could be combined with a sufficient proportion of CO_2_/steam during the material regeneration and coke removal stage in order to balance heat requirements. An industrial process making use of these cyclic reactions would either need to be operated in multiple fixed beds operating in sequence or in interconnected fluidized beds. For fixed beds, the evolution in kinetics might be problematic. On the other hand, the fact that partially reduced material has faster kinetics for methane conversion suggests that a well-mixed fluidized system might be advantageous, since a fraction of the particles in the reactor would always be partially reduced.

## 5 Conclusion

Thermodynamics predicts that Ca_2_Fe_2_O_5_ (C_2_F) is a promising metal oxide candidate to partially oxidize methane into CO/H_2_, owing to the low equilibrium *P*_O_2__ for its reduction. This also means it can be regenerated in steam or CO_2_ to generate H_2_ or CO. In chemical looping cycles, methane was partially oxidized by C_2_F to mainly CO and H_2_, with the CO yield ∼10 times higher than that of CO_2_.

The product of the reduction of Ca_2_Fe_2_O_5_ is metallic Fe (and CaO). This metallic Fe appears to play a significant role in driving catalytic pyrolysis and increasing the rate of oxygen transfer during the partial oxidation of methane by the oxygen carrier. The dehydrogenation of CH_4_ on the iron, which deposits carbon onto iron is likely to be the rate-determining step in the reduction of the oxygen carrier.

Once reduced to metallic iron, the oxygen carrier was an effective methane pyrolysis catalyst. Cycles which integrate partial oxidation and pyrolysis of methane in the chemical looping cycle offer a degree of flexibility in the heat balance and product ratios. The deposited carbon can be further gasified, while replenishing the reduced Ca_2_Fe_2_O_5_, under CO_2_, steam, and/or air depending on the desired product.

Rather than losing activity with cycles, the material activated. The initial induction period, which was attributed to the need to form sufficient metallic iron to catalyze the breakdown of methane, got shorter with cycling. Coking did not deactivate the material during the partial oxidation of methane, building up only after oxygen transfer was complete, and was readily removed during the oxidative regeneration before the next cycle. Once activated, the materials showed a stable performance over a reasonable number of cycles.

## 6 Data and Software Availability

All data for this work is provided within the paper, the associated Supporting Information, and on the repository https://www.repository.cam.ac.uk/.

## Supporting Information Available

Figure S1, schematic diagram of fluidized bed; Figure S2, DTG curve; Figure S3, temperature-programmed reduction in H_2_; Figure S4, blank experiments using 40 g of alumina sands; Figure S5, TPR in CH_4_ balance with N_2_; Table S1, CO yield in mol and its relation with C_2_F conversion; Figure S6, C_2_F conversion (%) for different reaction times during reduction in 5% H_2_/N_2_; Figure S7, off-gases concentration profile in percentage of methane pyrolysis cycle; Figure S8, syngas yield during methane pyrolysis; Figure S9, SEM and EDX results of fresh C_2_F; Figure S10, off-gases concentration profile; Figure S11, XRD patterns of fresh and after-cycled C_2_F; Figure S12, cycling experiments of reduction in CH_4_; and S10, data analysis (PDF)
