# Supplementary material for: Understanding the Behavior of Dicalcium Ferrite (Ca2Fe2O5) in Chemical Looping Syngas Production from CH4
Source: Energy Fuels. 2022 Aug 17;36(17):9410–22. doi: 10.1021/acs.energyfuels.2c01065 (PMC9442581; doi:10.1021/acs.energyfuels.2c01065)
Supplement: Supplementary file 1 — ef2c01065_si_001.pdf [file ef2c01065_si_001.pdf]

Supporting Information: Understanding the behaviour of dicalcium ferrite ($Ca_2Fe_2O_5$) in chemical looping syngas production from CH_4

Made Santihayu Sukma[a], *Yaoyao Zheng*[a], *Paul Hodgson*[a], *Stuart Ashley Scott*[a]*

[a]Department of Engineering, Trumpington Street, University of Cambridge, CB2 1PZ, United Kingdom

*Email: sas37@cam.ac.uk; Phone: (+44) 1223 3 32645

S1. Schematic diagram of the fluidised bed.

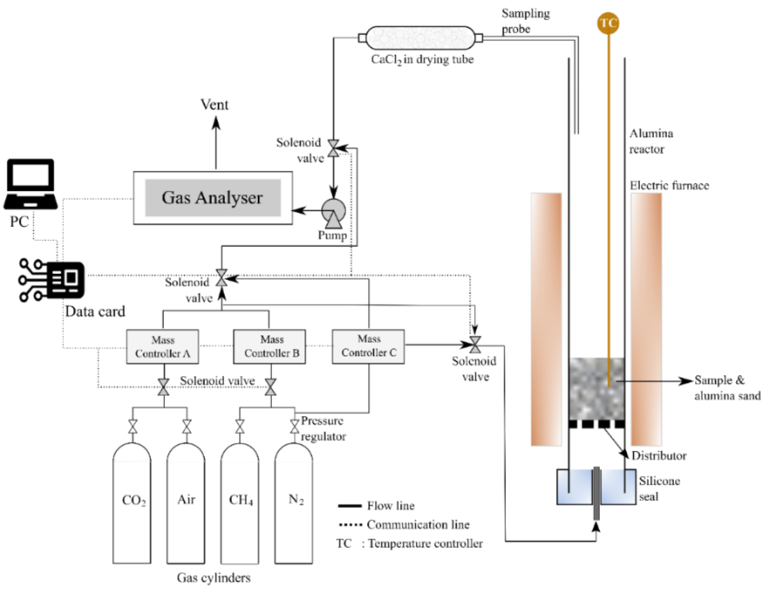

Figure S1. Schematic diagram of the fluidised bed.

S2. Temperature-programmed of Reduction in CH_4 and H_2 in the TGA.

Temperature-programmed of reduction (TPR) in H_2 was performed and the results are shown in **Fig. S2** and **S3**. Normalised mass of Fe_2O_3 and C2F during this TPR in H_2 is shown **Fig. S3**, and its derivative curve (DTG) in respected to operating temperature is shown in **Fig. S2**.

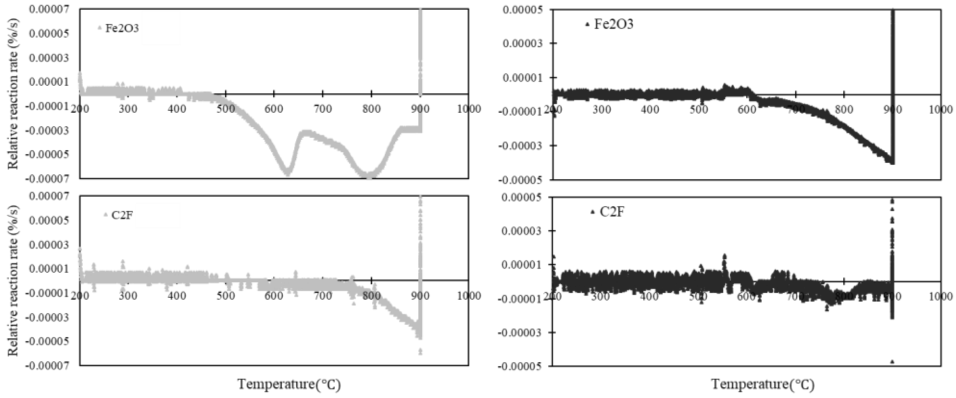

Figure S2. DTG curve: temperature *vs.* differential of mass change in %mass/s of Fe_2O_3 and $Ca_2Fe_2O_5$ during temperature-programmed reduction (TPR) from 200 – 900 °C at 10 °C/min and isothermal reduction at 900 °C for 120 mins in H_2 **(left**) and in CH_4 (**right**) balance with N_2 in the TGA.

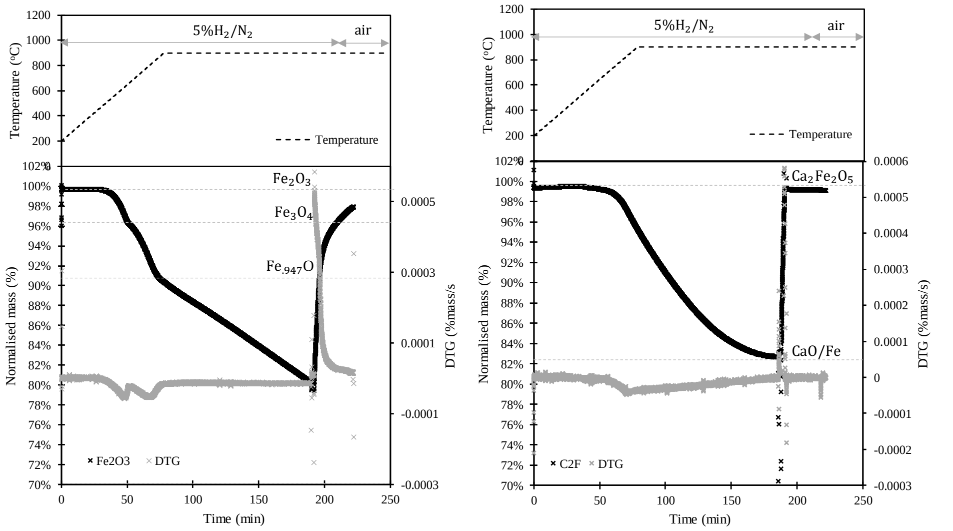

Figure S3. Temperature-programmed reduction in H_2: (i) ramping up temperature from 200 – 900 °C at 10 °C/min, (ii) holding it at 900 °C for 120 mins, and (iii) oxidising the reduced OCs in air at 900 °C (— normalised mass in %wt and — DTG curve).

S3. Blank Experiment in the Fluidised Bed.

A blank experiment was performed in a fluidised bed consisting of 40 grams of alumina sands in the alumina reactor with 5%CH_4/N_2 for 60 mins at 900 °C. The experimental conditions were the same as the fluidised bed experiments described in the paper (see **Section 2.3.**), except no C_2F was fed to the system. Molar flowrate of off gases is shown in **Fig. S4**.

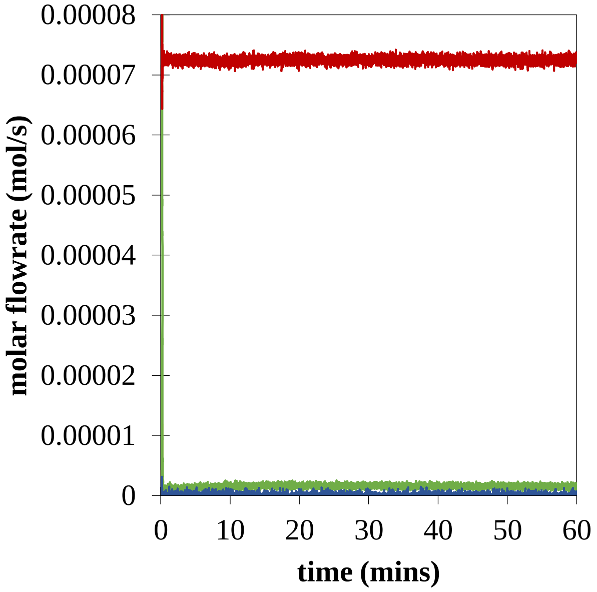

Figure S4. Blank experiments using 40 grams of alumina sands exposed in 5%CH_4/N_2 for 60 mins at 900 °C in the fluidised bed.

S4. Temperature-programme Reduction in CH_4 of Cycled C_2F.

After the C_2F was cycled for 37 times in the fluidised bed (results were shown in **Section 3.2**), the material was retrieved and tested in the TGA. Temperature-programme reduction in CH_4 was performed using this retrieved sample with similar steps as previously described (see **Section 2.2.**), except oxidation was completed only in air (CO_2 oxidation was omitted). Its normalised mass during the reduction in CH_4 was shown in **Fig. S5**.

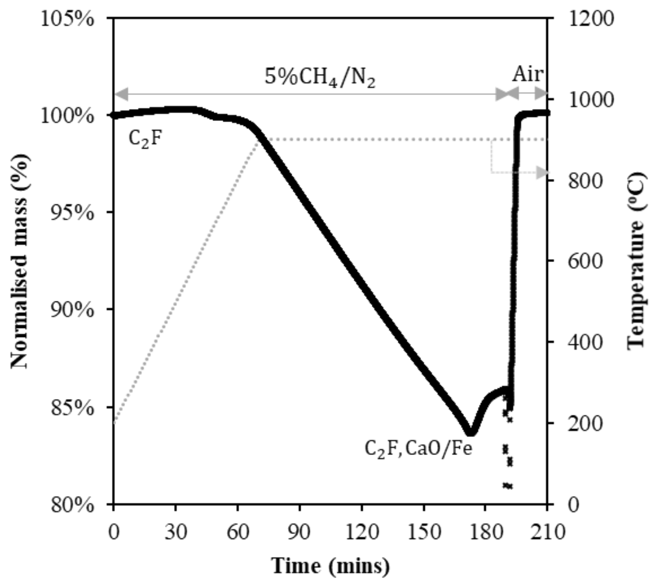

Figure S5. Temperature-programmed reduction (TPR) in CH_4 balance with N_2 in the TGA of the cycled-C_2F: the samples were heated from 200 to 900 °C at a heating rate of 10 °C/min and held for 120 mins in CH_4 balance with N_2 and followed by air oxidation for 20 mins at 900 °C.

S5. Reduction in 5%H_2/N_2 using C_2F in the Fluidised Bed.

The C_2F reduction in H_2 was performed in the fluidised bed as shown in **Fig. S1** and described in **Section 2.3**. In this particular experiment, 2 grams of the C_2F (500 – 850 μm, density of ~1500 kg/m^3) was fed into the alumina reactor filled with heated alumina sand (~40 grams) under N_2 at temperature of reaction. Reduction in H_2 was completed at 900 °C using 5% H_2 balance with N_2 and then the oxidation was completed in 20%CO_2 balance with N_2, hence CO was produced (see **Table S1**). The CO yield is used to calculate the %conversion (shown in **Fig. S6**) of the C_2F by comparing it with theoretical amount of CO that should be obtained if the C_2F is fully converted into CaO and metallic Fe. The reduction in H_2 was performed for different periods of time, i.e. 5, 10, 15, 20, 25 and 30 mins, while the oxidation duration was constant, i.e. 15 mins.

Table S1. CO yield in mol and its relation with the C_2F conversion

Max. CO yield (mol)		0.0088
Time (min)	CO yield (mol)	C_2F conversion*
0	0	0
5	0.0050	57%
10	0.0071	80%
15	0.0081	91%
20	0.0083	94%
30	0.0083	94%

*C_2F conversion was estimated by dividing CO yield (in mol) with the theoretical amount of CO yield if the C_2F is fully converted into metallic Fe and CaO.

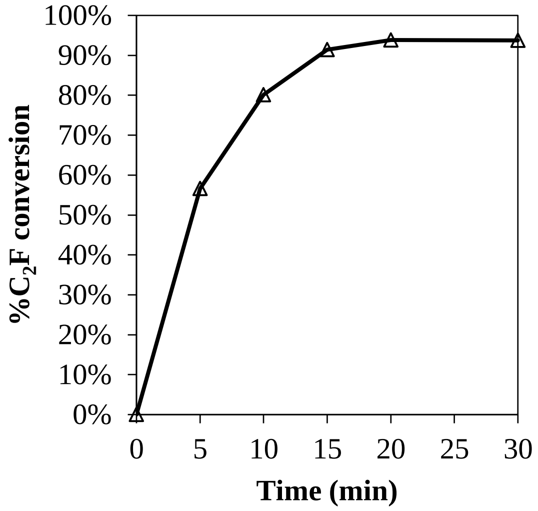

Figure S6. C_2F conversion (%) for different reaction times during reduction in 5%H_2/N_2 in the fluidised bed at 900 °C.

S6. The Reduced C_2F As A Methane Pyrolysis Catalyst.

The reduced C_2F's ability to catalyse methane pyrolysis was tested at different temperature, i.e. 700, 800, and 900 °C as described in **Section 3.3**. The experiments were performed in the

fluidised bed (see **Section 2.3**) through these following steps: (i) feeding gas with 5%H_2 balance with N_2 for 10 mins, (ii) reacting the mostly reduced C_2F with 5% CH_4 balance with N_2 for 60 mins, and finally (iii) oxidising it with 20%CO_2/N_2 for 20 mins. Off-gas profiles from these experiments at various temperature are shown in **Fig. S7** and corresponding yields are shown in **Fig. S8**.

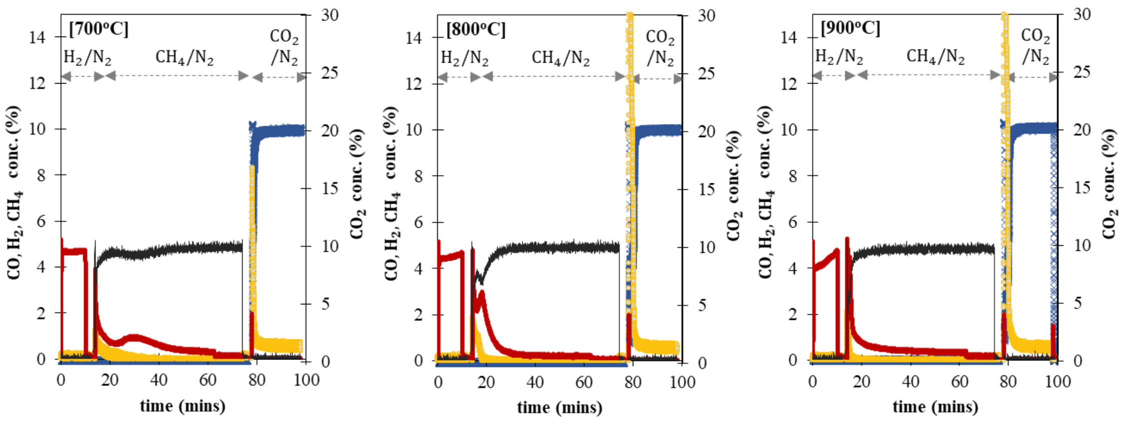

Figure S7. Off-gases concentration profile in percentage of methane pyrolysis cycle at 700, 800, and 900 °C.

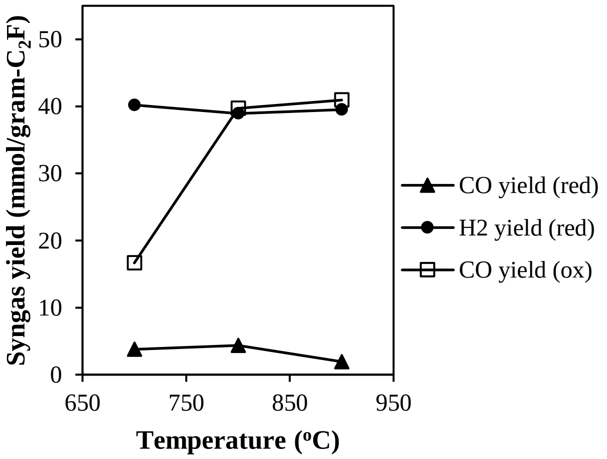

Figure S8. Syngas yield during methane pyrolysis: (i) 5%H_2/N_2 for 10 mins followed by (ii) 5%CH_4/N_2 for 60 mins and (iii) oxidation in 20%CO_2/N_2 for 20 mins at 700, 800 and 900 °C using partially reduced C_2F. CO (—▲—) and H_2 (—●—) yield (red) is CO and H_2 produced

when C_2F reacted with CH_4 in second stage, respectively and CO yield (ox) (—□—) is total CO produced during CO_2 oxidation stage.

S7. SEM and EDX Results.

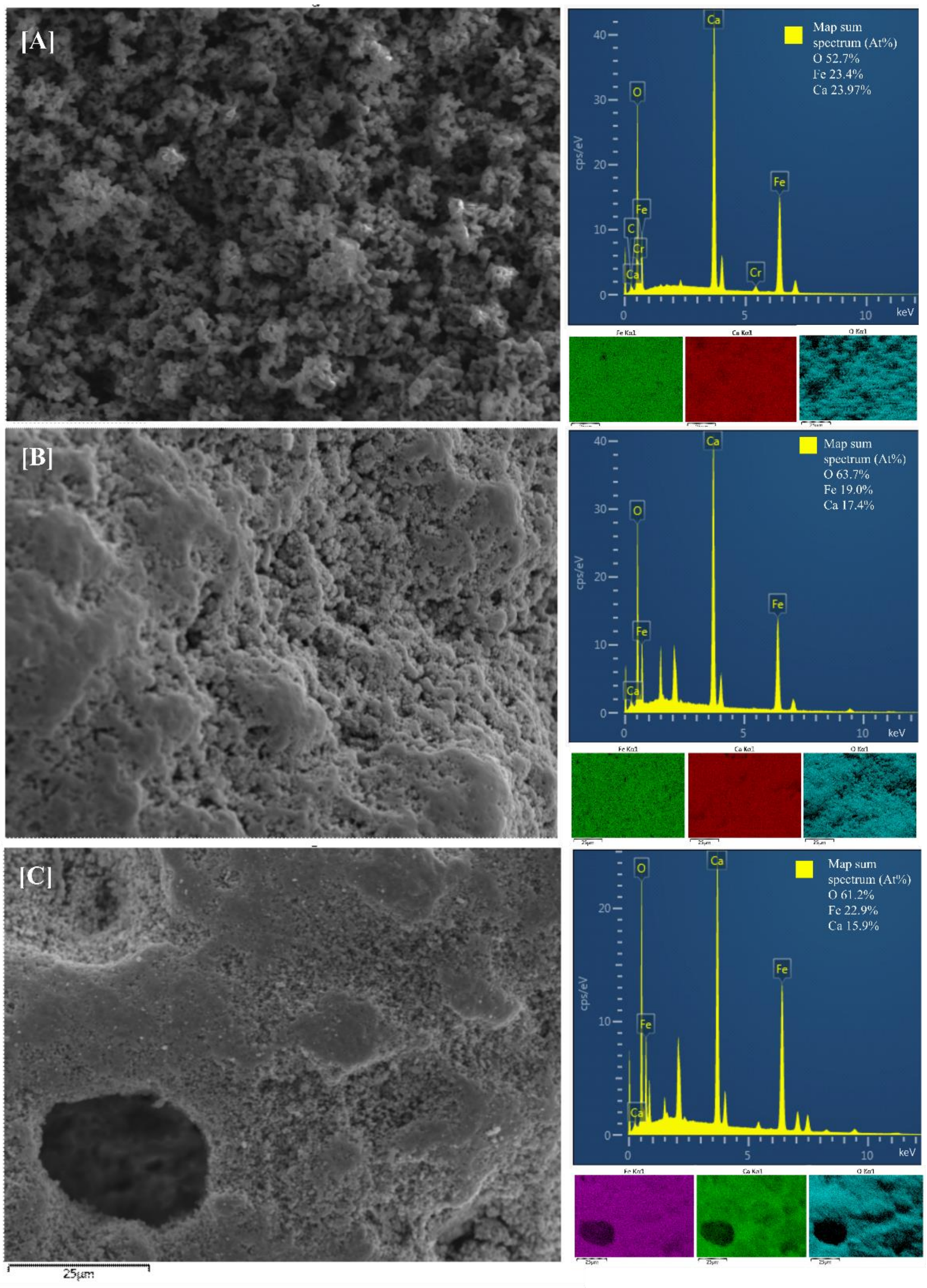

Figure S9. SEM and EDX results of fresh C_2F [**A**], after ~8 cycles [**B**] and at final cycle (cycle 37th) [**C**].

S8. Cyclic Redox Experiments in the Fluidised Bed at 900 °C using a Larger Amount of C2F

Similar cyclic redox experiments were performed for a larger batch of C2F. 2 grams of the materials were fed to the fluidised bed filled with heated ~40 grams of alumina sands under N_2 purging. Typical cycles as shown in **Fig. 5** was performed for 8 cycles at 900 °C and off-gases concentration profile is shown in **Fig. S10**. The materials were retrieved after 8 cycles and XRD was performed for this cycled material as shown in **Fig. S11**.

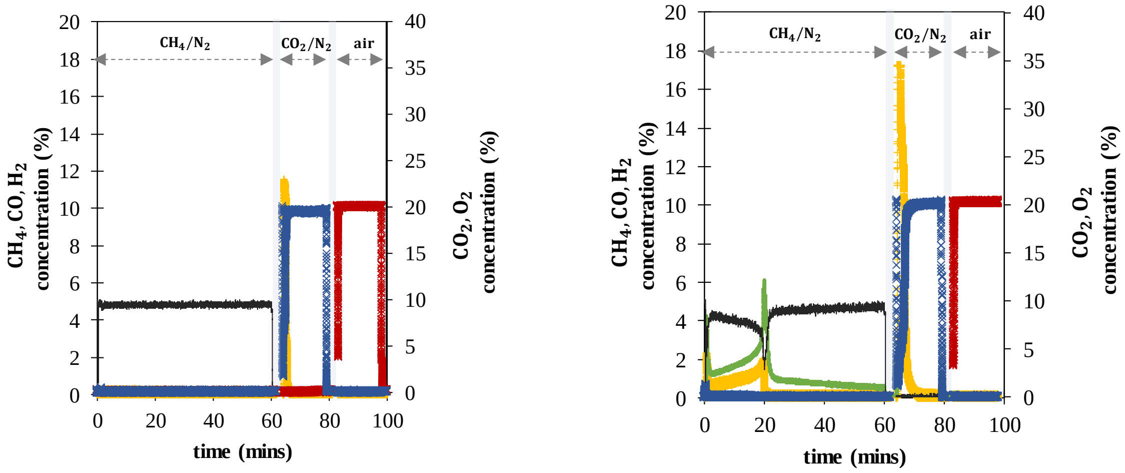

Figure S10. Off-gases concentration profile in percentage of redox experiments at 1st (**left**) and 7th (**right**) cycle at 900 °C. C2F was reacted in: 5%CH_4/N_2 for 60 mins, followed by oxidation in 20%CO_2/N_2 for 15 mins and in air for 15 mins.

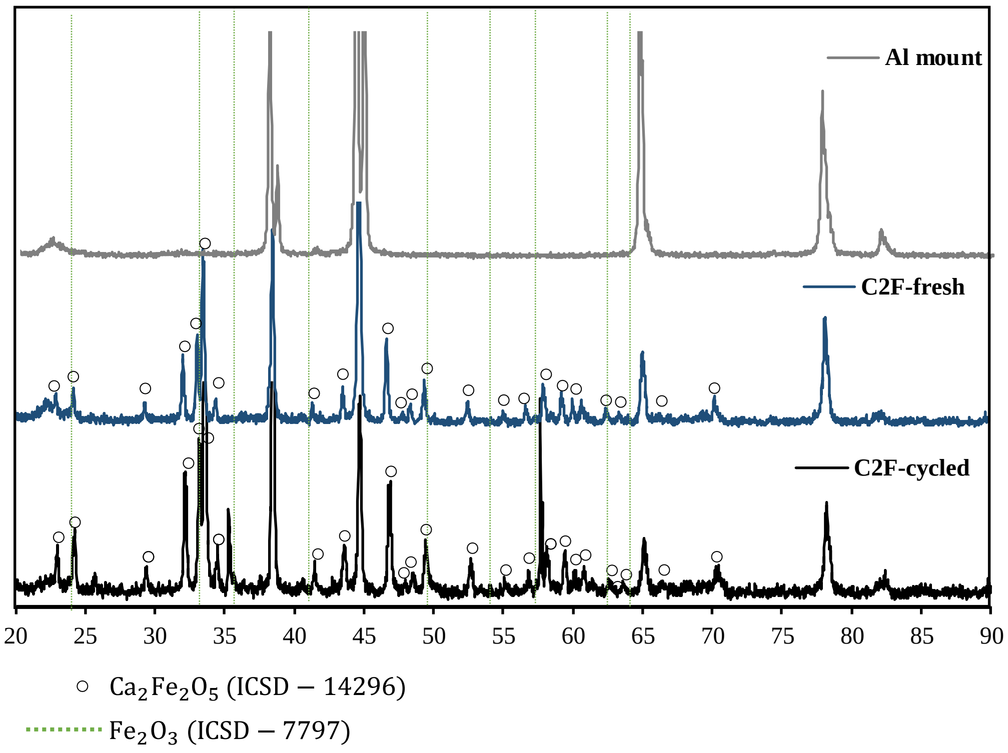

Figure S11. XRD patterns of the fresh and after-cycled C_2F. The cycling experiments were performed in the fluidised bed at 900 °C. The reference patterns were obtained from ICSD – 7797 for Fe_2O_3 and ICSD – 14296 for $Ca_2Fe_2O_5$. Those are labeled '---' and '○', respectively.

S9. Isothermal Cyclic Redox and Temperature-Programmed Reduction in CH_4 in the TGA.

Similar behaviour of the C_2F in the TGA and fluidised bed was observed, i.e. the C_2F required some cycles before it activated and readily reacted with the CH_4. TPR in CH_4 was performed after the C_2F initially was activated in cyclic redox experiments in the TGA as shown in **Fig. S12A**. The cyclic redox was performed for 4 cycles in which the C_2F was reduced in CH_4 at 900 °C for 2 hours before being replenished in air for 30 mins. It was then followed by the TPR in which: (i) the C_2F was heated up from 200 – 900 °C at a rate of 10°C/min and then (ii) held for 2 hours in CH_4 and finally (iii) it was oxidised in CO_2 and air for 90 and 15 mins at 900 °C. The normalised mass in % during the TPR is shown in **Fig. S12B**.

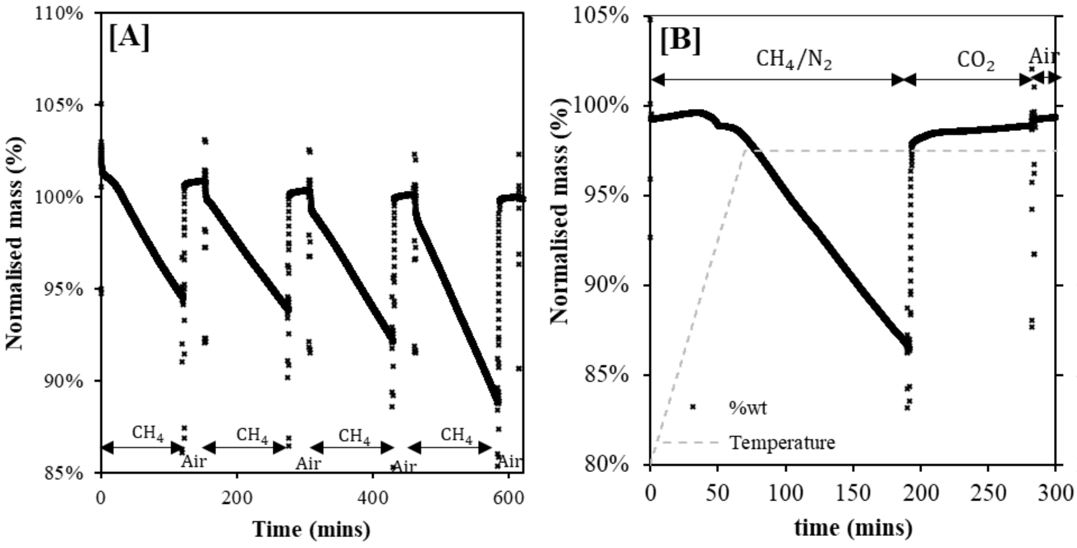

Figure S12. Cycling experiments of reduction in CH_4 for 2 hours and oxidation in air for 30 mins of the C_2F in the TGA at isothermal temperature of 900 °C [**A**] and normalised mass (%) during TPR in CH_4 from 200 – 900 °C at a rate of 10 °C/mins, followed by isothermal reduction in CH_4 for 2 hours and oxidation in CO_2 and air for 90 and 15 mins at 900 °C, respectively [**B**].

S10. Data Analysis.

In order to analyse the rates of oxygen transfer and conversion of methane, the molar flows of each product species must be determined. The gas analyser measures the composition on a dry basis, and in addition there is change in molar flow (albeit small as the feed is only 5% CH_4) that needs to be account for. Here, these corrections were made by assuming coke formation was negligible in which partial oxidation of methane dominated and some water might have been generated.

The concentrations of gases were measured dry basis, after the dryer tube filled with $CaCl_2$, by the ABB EL3020 gas analysers. During CH_4 reduction stage, the molar flowrate gas leaving the fluidised bed, excluding water (N_3) was computed using the carbon balance, as follows:

$$(z_{CH_4} + z_{CO} + z_{CO_2})N_3 = (x_{CH_4} + x_{CO} + x_{CO_2})N_1 \qquad \text{(S1)}$$

where the total molar flowrate of inlet gases (N_1) and its component composition (x_i) were known. The dry basis mole composition z_i is related to the mole fraction y_i by $y_iN_2 = z_iN_3$.

Here

z_i	:	molar fraction of outlet gases (dry)
N_3	:	total molar flowrate of outlet gases from the fluidised bed (dry-basis)
y_i	:	molar fraction of outlet gases from fluidised bed
N_2	:	total molar flowrate of outlet gases from fluidised bed
x_i	:	molar fraction of inlet gases to fluidised bed
N_1	:	total molar flowrate of inlet gases to fluidised bed

N_1 was estimated as follows:

$$N_1 = Q_N \times \rho_N \qquad \text{(S2)}$$

where

Q_N	:	volumetric flowrate into bed (at inlet conditions)
ρ_N	:	total molar density at inlet conditions

Steam produced (N_{H_2O}) was then estimated using hydrogen balance:

$$(4x_{CH_4} + 2x_{H_2})N_1 = 2N_{H_2O} + (4z_{CH_4} + 2z_{H_2})N_3$$

$$N_{H_2O} = \frac{(4x_{CH_4} + 2x_{H_2})N_1 - (4z_{CH_4} + 2z_{H_2})N_3}{2} \qquad \text{(S3)}$$

Accordingly, total molar flowrate of product outlet, N_2 is:

$$N_2 = N_{H_2O} + N_3 \qquad \text{(S4)}$$

Molar flowrate in mol/s for each product gas streams was calculated as follows:

$$n_i = y_iN_2 = z_iN_3 \qquad \text{(S5)}$$

and yield of product during time period, t, was calculated by integrating area under molar flowrate *vs.* time curve as follows:

$$\sum n_i = \int_{t=0}^{t} y_i N_2 \, dt \quad \text{(S6)}$$

For comparison, N_3 could also be estimated, assuming no steam is generated during the reduction. Thus, carbon balance becomes:

$$\left(z_{CH_4} + z_{CO} + z_{CO_2}\right)N_3 + N_{coke} = \left(x_{CH_4} + x_{CO} + x_{CO_2}\right)N_1 \quad \text{(S7)}$$

where N_{coke} is the rate of coke deposition. N_3 can be predicted using hydrogen balance now by assuming $N_{H_2O} = 0$, i.e.

$$N_3 = \frac{\left(4x_{CH_4} + 2x_{H_2}\right)N_1}{\left(4z_{CH_4} + 2z_{H_2}\right)} \quad \text{(S8)}$$

Accordingly, molar flowrate can be simply calculated:

$$n_i = z_i N_3 \quad \text{(S9)}$$

The results given in the paper use the former method, i.e. ignoring coking, and should be most accurate during the initial partial oxidation phase. When there is no oxygen transfer and only coking occurs, the latter method should instead be better. However, comparing these two methods to estimate carbon balance for cycle 7th as an example case gave error of less than 10% of product gas yields (i.e. CO, H_2 and CO_2) during the reduction stage. This is to be expected since the system is dilute and the conversion of the 5% methane is not complete; the extent of correction for changes in molar flow rate is small, and in practice could be probably be neglected entirely.

During oxidation in CO_2, there was no H_2O produced, hence $z_i = y_i$, whereas the inlet gases remain constant, i.e. 20% CO_2 balance with nitrogen at 2 liter per min. The total molar flowrate of the outlet gases (N_3) was predicted using nitrogen balance as follow:

$$x_{N_2}N_1 = z_{N_2}N_3 = \left(1 - z_{CO} - z_{CO_2}\right)N_3 \quad \text{(S10)}$$

In this step, CO can also be produced from coke gasification, i.e. $C_{(s)} + CO_{2(g)} \leftrightarrow 2CO_{(g)}$ in addition to the CO produced due to CO_2 oxidation of the C_2F. The rate of coke gasification (N_{coke}) can be derived using carbon balance:

$$x_{CO_2}N_1 + N_{coke} = (z_{CO_2} + z_{CO})N_3 \tag{S11}$$

This allows an estimate of the coke produced during the previous reduction.

Metal oxide conversion was defined as the total mass of oxygen lattice being transferred to react with fuel compared with to the mass loss obtained when the materials are fully reduced:

$$x_{metox} = \frac{m_{O_2}}{m_{O_2,max}} \tag{S12}$$

where

x_{metox} : metal oxides conversion (%)

m_{O_2} : amount of oxygen released during methane reduction per mass of oxygen carrier.

$m_{O_2,max}$: maximum oxygen contained in oxygen carriers (gram/gram), i.e. $m_{O_2,max}$ of C_2F and Fe_2O_3 is 17.7%wt and ~30%wt.
